# Towards discovery of inhibitors of the undecaprenyl-pyrophosphate phosphatase BacA by virtual high-throughput screening

**DOI:** 10.1016/j.csbj.2022.05.010

**Published:** 2022-05-11

**Authors:** Marko Jukič, Rodolphe Auger, Victor Folcher, Matic Proj, Hélène Barreteau, Stanislav Gobec, Thierry Touzé

**Affiliations:** a University of Maribor, Faculty of Chemistry and Chemical Engineering, Laboratory of Physical Chemistry and Chemical Thermodynamics, Smetanova 17, Maribor SI-2000, Slovenia; b University of Primorska, Faculty of Mathematics, Natural Sciences and Information Technologies, Glagoljaška 8, Koper SI-6000, Slovenia; c Université Paris-Saclay, CEA, CNRS, Institute for Integrative Biology of the Cell (I2BC), Gif-sur-Yvette FR-91198, France; d Univerza v Ljubljani, Fakulteta za Farmacijo, Aškerčeva cesta 7, Ljubljana SI-1000, Slovenia

**Keywords:** C_15_-PP, Farnesyl pyrophosphate, C_5_-PP, Isopentenyl pyrophosphate, C_55_-P, Undecaprenyl phosphate, C_55_-PP, Undecaprenyl pyrophosphate, GlcNAc, *N*-acetylglucosamine, HTVS, High-Throughput Virtual Screening, MD, Molecular Dynamics, MIC, Minimum Inhibitory Concentration, MurNAc, *N*-acetylmuramic acid, PBPs, Penicillin-binding proteins, PG, Peptidoglycan, PP, Pyrophosphate, RA, Residual activity, RMSD, Root-mean-square deviation, TLC, Thin layer chromatography, VS, Virtual Screening, BacA, Undecaprenyl pyrophosphate phosphatase, Escherichia coli, Antibacterials, Antibacterial drug design, Binding site identification, Virtual screening, Non-covalent inhibitors, In silico drug design, Molecular dynamics, Ensemble docking

## Abstract

Increasing resistance to common antibiotics is becoming a major challenge that requires the development of new antibacterial agents. Peptidoglycan is an essential heteropolymer of the bacterial envelope that ensures the integrity and shape of all bacteria and is also an important target for antibiotics. The biosynthesis of peptidoglycan depends on a lipid carrier, undecaprenyl phosphate. As a byproduct of peptidoglycan polymerization, the lipid carrier is released as undecaprenyl pyrophosphate, which must be recycled to allow new polymerization cycles. To this end, it undergoes a dephosphorylation process catalyzed by the membrane phosphatase BacA, which is specific and highly conserved in bacteria. In the present study, we identified small molecules displaying inhibitory potency towards BacA. We began by preparing a commercial compound library, followed by high-throughput virtual screening by ensemble docking using the 3D structure of BacA and molecular dynamics snapshots to account for the flexibility of the protein. Of 83 compounds computationally selected and tested in a biochemical assay, one sulfamoylthiophene molecule showed significant inhibition of the undecaprenyl pyrophosphate dephosphorylation activity catalyzed by BacA. Subsequently, an additional 33 scaffold analogs were selected and acquired, of which 6 compounds exhibited BacA inhibition. The IC_50_ values of these compounds ranged from 42 to 366 μM. In addition, significant antibacterial activity against *Escherichia coli* was observed in TolC/PAP2-depleted strains. We believe that the overall strategy followed in this study and the identified class of inhibitors provide a solid foundation for the further development of potent BacA-targeted inhibitors and the discovery of novel antibacterial compounds.

## Highlights

• BacA is an important conserved bacterial protein and a promising drug target.
• We identified the first small molecule inhibitors of BacA *via* ensemble docking.
• Novel inhibitors possess 5-sulfamoyl-2-thenoic acid scaffold.
• Hit compounds display IC_50_s from 42 to 374 μM and antibacterial activity on *E. coli.*
• Reported compounds are a valuable starting point for new antibacterial drug design.

## Graphical abstract

**Figure:**
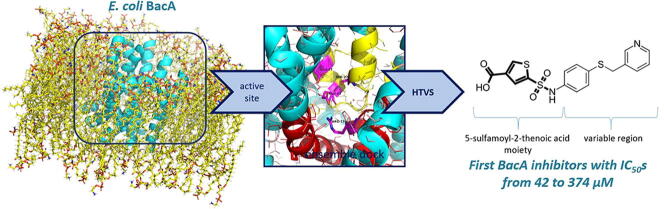

## 1 Introduction

**Fig. 1:**
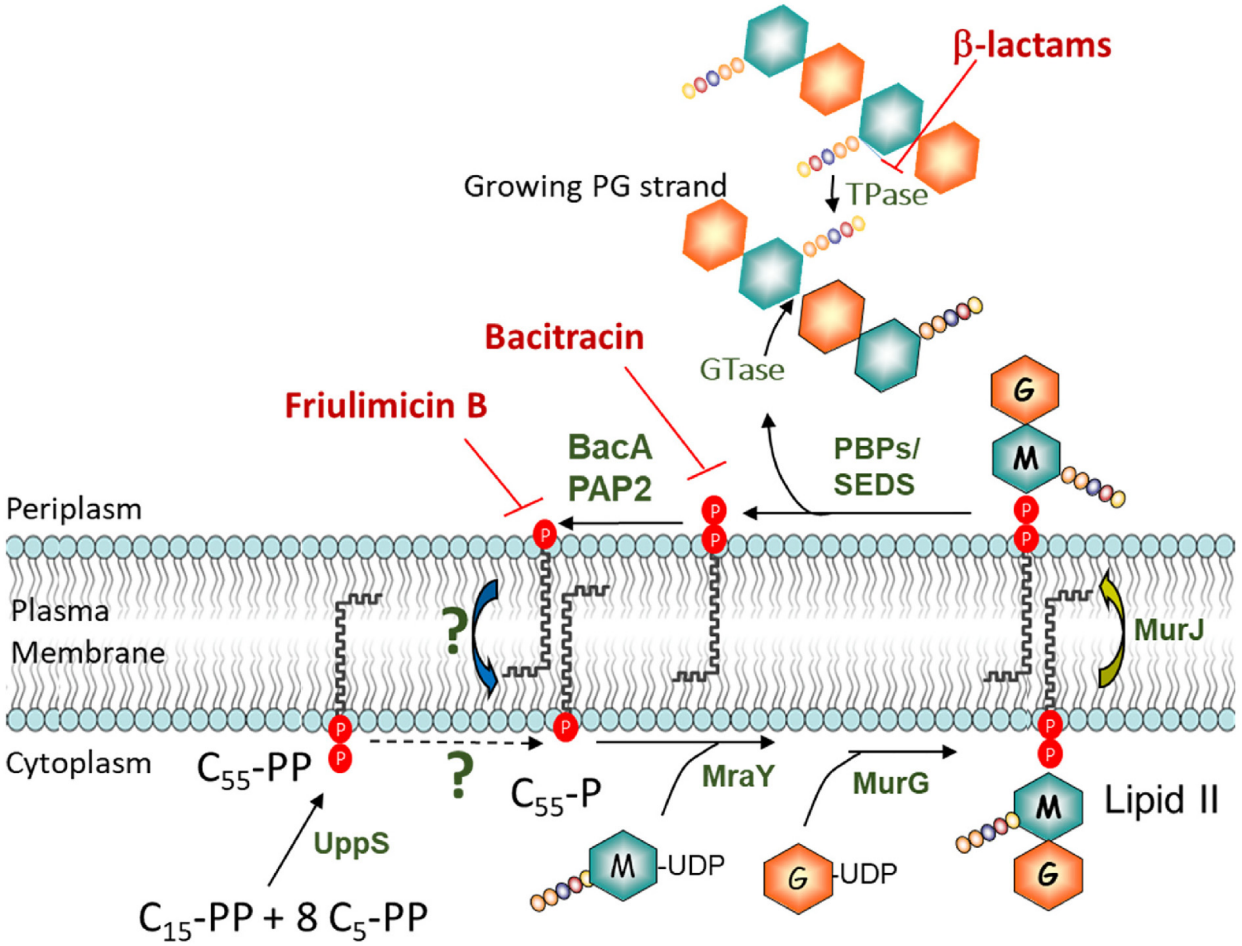
De novo synthesis, use and recycling of C_55_-P and peptidoglycan synthesis. At the cytoplasmic side of the membrane, the enzyme UppS catalyses de novo synthesis of C_55_-PP, which is subsequently converted to C_55_-P by dephosphorylation by a yet unknown enzyme. The successive transfer of phospho-MurNAc(pentapeptide) and GlcNAc to C_55_-P is catalysed by MraY and MurG, respectively, yielding lipid II. The lipid carrier, with the help of the flippase MurJ, allows the translocation of the glycan-pentapeptide subunit to the periplasmic side of the membrane, where it is eventually transferred to the growing peptidoglycan through glycosyltransferase activity (GTase) of PBPs or SEDS enzymes. The glycan strands are then reticulated by transpeptidase activities (TPase) of PBPs. Finally, the lipid carrier is released in its pyrophosphate form, C_55_-PP, which is dephosphorylated by a C_55_-PP phosphatase of BacA or PAP2 families yielding C_55_-P. The latter is then flipped back to the cytoplasmic side by a yet unknown mechanism to be reused for another cycle of transfer of a peptidoglycan subunit. M = MurNAc, G = GlcNAc, P = phosphate. Adapted from Manat et al., 2014 [7].

Peptidoglycan (PG) constitutes the essential and specific exoskeleton of a vast majority of bacterial cells. It is also one of their weaknesses, which is underlined by the extent of antibacterial compounds that impede its biogenesis, maturation, and regulation. PG surrounds the plasma membrane of the cell, where it withstands the internal osmotic pressure, confers the cell shape and serves as a scaffold for anchoring other cell envelope components. It constitutes a complex heteropolymer made of long glycan chains with alternating *N*-acetylglucosamine (GlcNAC) and *N*-acetylmuramic acid (MurNAc) residues, which are cross-linked by short peptide stems [1]. The PG subunit consists of a disaccharide-pentapeptide that is sequentially assembled on a lipid carrier, the undecaprenyl phosphate (C_55_-P), on the cytoplasmic side of the plasma membrane, yielding lipid II (undecaprenyl-pyrophosphoryl-MurNAc(peptide)-GlcNAc) (Fig. 1) [2]. Anchoring of the PG subunit to C_55_-P allows its translocation to the outer side of the plasma membrane by the MurJ flippase [3]. Afterwards, the disaccharide-peptide subunit is transferred to the growing end of peptidoglycan strands by penicillin-binding proteins (PBPs) [4] or SEDS (shape, elongation, division, and sporulation) proteins [5], releasing the lipid carrier as undecaprenyl pyrophosphate (C_55_-PP) at the outer leaflet of the membrane (Fig. 1). C_55_-P must be recycled to fuel the high polymerization rate of PG since it is present in a low copy number per cell and is also used for the synthesis of other cell envelope polymers [[6], [7]]. Therefore, the rate of C_55_-P recycling is critical to ensure the proper turnover of cell envelope biogenesis. The recycling of C_55_-P requires the dephosphorylation of C_55_-PP and the flip of C_55_-P back to the inner side of the membrane. Two classes of transmembrane C_55_-PP phosphatases have been identified: BacA enzymes and a sub-group of PAP2 enzymes (type 2 phosphatidic acid phosphatases) [[8], [9]]. *Escherichia coli* has one BacA and three PAP2 (PgpB, YbjG, and LpxT) proteins. From their structures, it was inferred that the two classes of C_55_-PP phosphatases bind their substrate from the outer leaflet of the plasma membrane, i.e., the C_55_-PP released by PG polymerases [[10], [11], [12], [13]]. PgpB and BacA were found to interact with the PG polymerase PBP1B and to stimulate its glycosyltransferase activity [14]. C_55_-P recycling is hampered by the antibiotics bacitracin and friulimycin B, which sequester C_55_-PP and C_55_-P, respectively, thus blocking PG biosynthesis and causing cell lysis. These observations demonstrate the importance of C_55_-P recycling and its attractiveness as a target for new drugs.

BacA is an important and conserved protein in the bacterial world and thus can be considered a promising drug target. To identify BacA-specific inhibitors, a high-throughput virtual screening was performed followed by biological evaluation of hit compounds. The in silico screening method used ensemble molecular docking and molecular dynamics (MD) on the available crystal structure of the apo-form of BacA (PDB ID: 5OON) [12] to evaluate the immediate conformational flexibility of the protein. The virtual screening scenario led to the selection of a total of 116 different compounds with characteristic functional groups. We then identified 7 compounds with a common 5-sulfamoyl-2-thenoic acid scaffold that exhibited inhibitory activity against BacA and BacA-dependent antibacterial activity.

### 1.1 3D structure and function of BacA

The 3D structure of BacA reveals key structural motifs that are common to a variety of transporters, which suggests that BacA, besides its C_55_-PP phosphatase activity, may also catalyse the flip of C_55_-P. In contrast to the dephosphorylation step, the subsequent flip of C_55_-P is still a black box in C_55_-P recycling ([[12], [13]], Fig. 1). In *E. coli*, BacA is responsible for 75% of the C_55_-PP phosphatase activity [9]. Due to the plurality of C_55_-PP phosphatases, the deletion of the *bacA* together with *pgpB* and *ybjG* genes was required to get a lethal phenotype [9]. Nevertheless, the deletion of *bacA* in *Streptococcus pneumoniae*, *Staphylococcus aureus,* and *Mycobacterium smegmatis* abolishes their virulence or their ability to form biofilms [[15], [16]]. Furthermore, the BacA proteins of *Lactococcus lactis* and *Enterococcus faecalis* are targeted by bacteriocins that cause membrane leakage by yet unknown mechanisms [17].

**Fig. 2:**
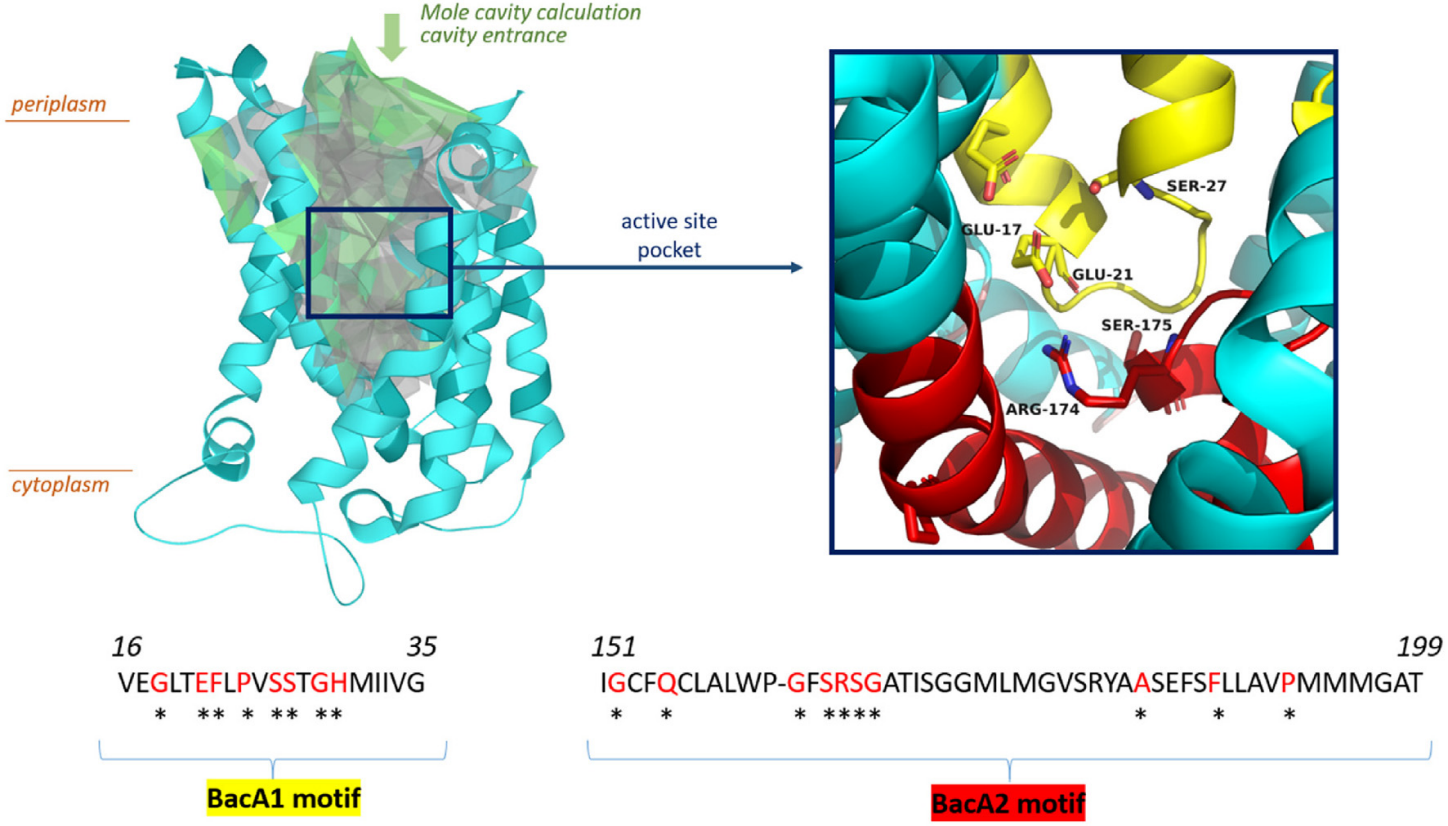
Crystal structure of BacA from *E. coli*. Top: view of BacA from the membrane plane (left). The structure is shown as a cartoon representation in cyan, with the largest cavity of the protein highlighted in green-grey, as calculated by the Mole software, and the entrance of the periplasmic cavity highlighted by a green arrow. The proposed binding pocket for C_55_-PP is shown in a box (right), with the conserved motifs, designated BacA1 and BacA2, coloured yellow and red, respectively, and the rest of the protein coloured cyan. The active site residues Glu17, Glu21, Ser27, Arg174, and Ser175 are shown in sticks and coloured according to the motifs. Bottom: the sequence of the BacA1 and BacA2 motifs, with the conserved residues in red and marked with asterisks. (For interpretation of the references to colour in this figure legend, the reader is referred to the web version of this article.)

BacA consists of two pseudosymmetric domains, which are in an inverted orientation to one another with respect to the membrane plane [[12], [13]]. Each domain contains three transmembrane helices and a pair of short, antiparallel re-entrant helices. In each pair, the re-entrant helices are connected by a short loop enclosing one of the conserved motifs, designated as BacA1 and BacA2 (Fig. 2). These motifs come in close proximity to each other at mid-section of the protein in the mid-plane of the membrane (Fig. 2). The enzyme is open to the outside and to the periplasmic leaflet by a cavity that extends half-way into the membrane, where BacA1 and BacA2 motifs meet each other to shape the bottom of the cavity. The size of the cavity was calculated by the Mole software as the largest cavity 1 with a volume of 4628 Å^3^ (probe radius 5, interior threshold 1.1) and is shown in green-yellow in Fig. 2 [18]. The hydrolysis of C_55_-PP may therefore take place at the latter position. The conserved residues Glu21 and Ser27 from BacA1 motif and Arg174 from BacA2 motif were shown to be central for C_55_-PP catalysis [19]. Ser27 is a strictly conserved residue, which upon mutagenesis to Ala abolishes the phosphatase activity of BacA. It was then proposed to be responsible for the nucleophilic attack at the β-phosphate group of C_55_-PP.

## 2 Material and methods

### 2.1 Molecular library preparation

**Fig. 3:**
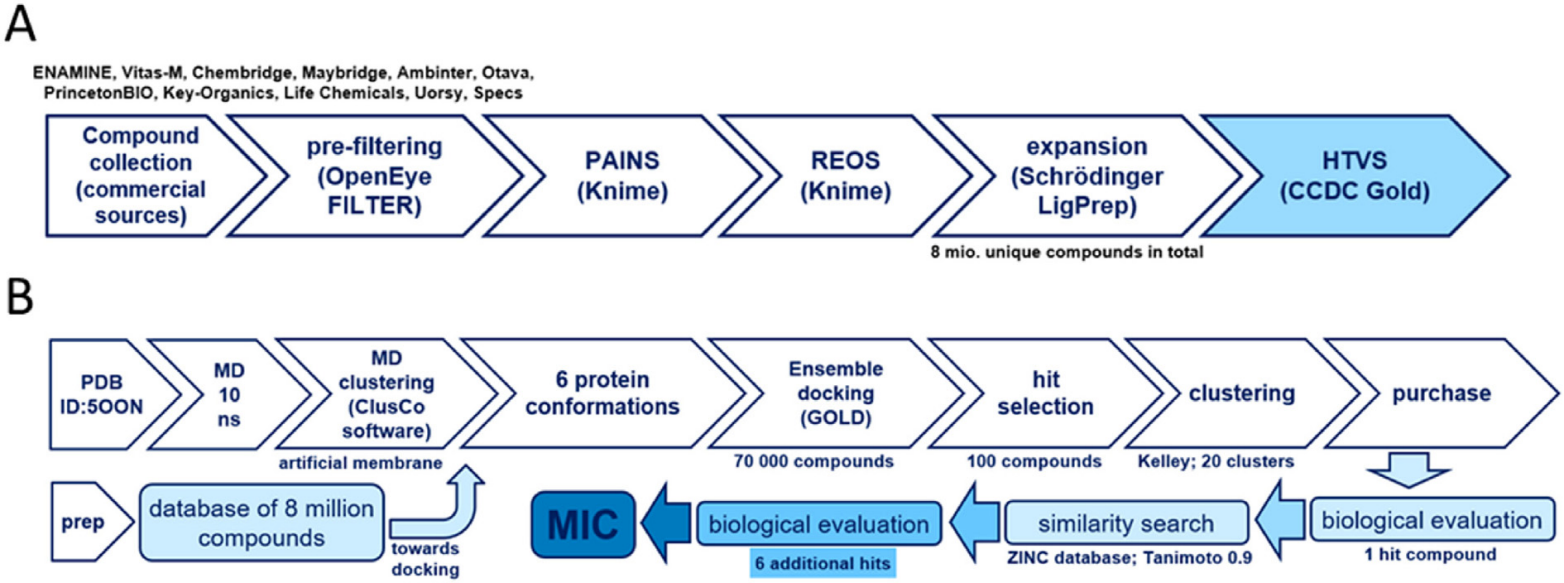
(A) Used workflow for molecular library preparation; (B). High-throughput virtual screening on the *E. coli* BacA protein and biological evaluation.

Library preparation for HTVS (high-throughput virtual screening) is an essential step for efficient downstream processing. We commenced with conglomeration of commercial compound databases from ENAMINE, Vitas-M, Chembridge, Maybridge, Ambinter, Otava, PrincetonBIO, Key-Organics, Life Chemicals, Uorsy, and Specs suppliers to gather 6,836,270 compounds in total. We then proceeded towards pre-filtering using OpenEye FILTER software (FILTER 2.5.1.4 version, OpenEye Scientific Software, Inc., Santa Fe, NM, USA; https://www.eyesopen.com). The goal was exclusion of structural faults, metals, small fragments, extra-large molecules, aggregators, and compounds with poor physico-chemical properties, e.g., solubility. The following parameters were applied: min_molwt 250, max_molwt 800, min_solubility poorly, allowed elements (H, C, N, O, F, P, S, Cl, Br, I, Si). Furthermore, known or predicted aggregators were removed with FILTER software (aggregators true “eliminate known aggregators” and pred_agg true “eliminate predicted aggregators”) while the library was filtered for PAINS and REOS structures using inhouse KNIME workflows with RDKit nodes [[20], [21], [22]]. In this manner, all structures in the library were compared to the selection of SMARTS-formatted flags and removed accordingly to the filtered library consisting of 3,731,096 compounds. After initial compound collection, library was expanded to 8 million unique compounds via final enumeration of undefined chiral centres, calculation of tautomeric structures, ionisation at pH 7.4 and minimisation (using OPLS3 force-field) towards the final 3D conformation [23]. The final step was performed using command line Ligprep software by Schrödinger (Release 2019–1, Schrödinger, LLC, New York, NY, USA; [[24], [25]] as illustrated in Fig. 3, panel A.

### 2.2 Molecular dynamics

Crystal complex (PDB ID: 5OON) [12] was prepared with Yasara software [26]. Missing hydrogens were added, overlaps adjusted, hydrogen bond network optimized, and residue ionisation assigned at pH 7.4 (cleanup, 270 residues, 29.428 kDa; [[27], [28]]. The protein was placed (based on the most exposed hydrophobic residues) in an artificial membrane model consisting of 100% phosphatidyl-ethanolamine-164 molecules and solvated using TIP3P water model (cubic; 15 Å padding – 80.82×78.72×80.82 Å, periodic boundary conditions). To neutralize the cell, a physiological concentration of 0.9% NaCl was added with an appropriate excess of either Na^+^ (24) or Cl^−^ (28). After steepest descent and simulated annealing minimizations with equilibration (250 ps) to remove steric clashes, the MD production run of 5 ns was calculated using the AMBER14 force field for the solute, GAFF and AM1BCC for ligands as well as TIP3P for water [29]. Nonbonded long-range interactions were calculated with the Particle Mesh Ewald algorithm. Timestep of 1.25 fs for bonded interactions and 2.5 fs for non-bonded interactions was used at a temperature of 298 K and a pressure of 1 atm (NPT ensemble; densostat) with snapshots saved every 100 ps. No SHAKE was used. Energy parameters of the system were stable throughout the production run, as were root-mean-square deviation (RMSD) values of the protein backbone.

### 2.3 High throughput virtual screening

Protein conformation model snapshots were superimposed on the starting structure and clustered using ClusCo software (hierarchical clustering, pairwise average-linkage manner, RMSD score) [30]. Ensemble docking experiment was performed using GOLD (CCDC Enterprise; 5.5 version; [31]. Five protein structures obtained from clustered (ClusCo) MD trajectory were aligned and superimposed to the first structure (crystal complex PDB ID: 5OON) and hydrogens added using Hermes software (CCDC Enterprise; 5.5 version). Structures were also checked and optimized using Protein Preparation by Schrödinger (Release 2019-1, Schrödinger, LLC, New York, NY, USA). Asn, Gln and His tautomers from the MD calculation were used. Glu17 and Glu49 were predicted to have potential to be protonated, however, since both residues are located out of the binding site, their protonation state has no effect on docking. Heavy atoms (2 Hg), waters (6 molecules, bulk), 2-amino-2-hydroxymethyl-propane-1,3-diol (TRS, 1 molecule), and (2*R*)-2,3-dihydroxypropyl (9*Z*)-octadec-9-enoate (OLS, 2 molecules) were removed as they were nonspecifically bound along the hydrophobic outer surface of the protein. The binding site was defined as a 12 Å region around Ser27 at the catalytic site as described by Manat et al. [19]. Flood fill settings with radius 12, origin 49.363, 42.159, 42.173, do_cavity 1, floodfill_atom_no 0, and floodfill_center point parameters were used. All planar R-NR_1_R_2_ were able to flip as well as protonated carboxylic acids. Torsion angle distributions and rotatable bond postprocessing were set to the default. Docking was performed with PLP scoring function with early termination (3 solutions, rms 1.5) enabled and default GOLD parameter file used [32]. Docking scores were normalized via N^1/3^ to abolish large molecule-bias and identify druglike compounds [33]. Genetic algorithm settings were set at ensemble with autoscale at 0.3 for first screening and 1 for final screening. Parallel gold calculation (initial database of 8 million compounds was trenched into 14 parts to alleviate parallel calculations on a cluster) was performed with concatenation of results and retention of best binding poses. No constraints were used in docking experiment. The results were analyzed using Data Warrior software and sorted according to the normalized GOLD PLP Fitness score. The top-scoring compounds were purchased from several vendors and evaluated biochemically and microbiologically. The workflow used for HTVS is summarized in Fig. 3, panel B.

### 2.4 Chemicals

Isopentenyl pyrophosphate (C_5_-PP) and farnesyl pyrophosphate (C_15_-PP) were purchased from Sigma. [^14^C]C_5_-PP (50.6 mCi/mmol) was purchased from Perkin Elmer Life Sciences. The [^14^C]C_55_-PP substrate was prepared as described below by successive condensations of [^14^C]C_5_-PP onto C_15_-PP catalysed by the purified UppS enzyme. The *n*-dodecyl-β-D-maltopyranoside (DDM) detergent was purchased from Anatrace and nickel-nitrilotriacetate-agarose (Ni^2+^-NTA-agarose) was from Qiagen. Compounds were purchased from Enamine and ChemBridge, and their identity and purity were confirmed (see Supplementary Information). All other materials were reagent grade and obtained from commercial sources.

### 2.5 Analytics

^1^H NMR spectra for active compounds were recorded in a deuterated solvent on a Bruker Avance III 400 MHz spectrometer, operating at 400 MHz frequency. HRMS mass spectra were recorded using a Thermo Scientific Q Exactive Plus mass spectrometer. Analytical reversed-phase HPLC was performed on Thermo Scientific Dionex UltiMate 3000 UHPLC system equipped with an Acquity UPLC® BEH Phenyl Column (details and full analytics can be found in Supplementary Information).

### 2.6 Bacterial strains, plasmids and media

All *E. coli* strains and plasmids used are listed in Table 1. Bacteria were grown in 2YT broth supplemented, when required, with ampicillin or kanamycin at concentrations of 100 and 50 µg/ml, respectively. The BWΔ*tolC* strain was obtained from the Keio collection. The BWΔ*bacA*Δ*tolC* and BW*bacA*-singleΔ*tolC* were generated by P1 transduction of the Δ*tolC*::Kan^R^ cassette from BWΔ*tolC* strain to BWΔ*bacA* and DMEG9 recipient strains, respectively. The strains *S. aureus* RN4220 and *S. pneumoniae* R6 were used to evaluate the antibacterial activity of certain compounds. *S. aureus* was grown on BHI (Brain Heart Infusion) broth and *S. pneumoniae* was grown on Colombia broth supplemented with 5% horse blood.

**Table 1:** All strains and plasmids used in our work.

Strains	Genotype	Source
DH5α	*supE44, lacU169, hsdR17, recA1, endA1, gyr696, relA1, 80d lacZ*Δ*M15*	Invitrogen
C43(DE3)	*F-ompT, gal, hsdsB (r_BnB_), dcm, DE3*	Avidis
BW25113	*lacI*^q^*rrnB*_T14_ Δ*lacZ*_WJ16,_*hsdR*514 Δ*ara BAD*_AH33_ Δ*rhaBAD*_LD78_	[34]
DMEG9	BW25113 Δ*ybjG*, Δ*lpxT*, Δ*pgpB*	[9]
BWΔ*bacA*	BW25113 Δ*bacA*	[35]
BWΔ*tolC*	BW25113 Δ*tolC*::Kan^R^	[36]
BWΔ*bacA*Δ*tolC*	BW25113 Δ*bacA,* Δ*tolC*::Kan^R^	This study
BW*bacA*-singleΔ*tolC*	BW25113 Δ*ybjG*, Δ*lpxT*, Δ*pgpB,* Δ*tolC*::Kan^R^	This study

Plasmids		
pTRCH30		[37]
p*Trc*Bac30	p*Trc*H30: *bacA E. coli*	[8]
p*Trc*H60pgpB	p*Trc*H60: *pgpB E.coli*	[35]

### 2.7 Purification of BacA and PgpB

C43(DE3) cells carrying p*Trc*Bac30 plasmid were used for the expression of *N*-terminally His_6_-tagged BacA (His_6_-BacA) protein from *E. coli*. Cells were grown in 2YT-ampicillin at 37 °C and when the OD_600_ reached 0.9, the temperature of the culture was decreased to 22 °C and protein expression was induced by addition of 1 mM IPTG. Incubation was continued overnight at 22 °C and bacteria were harvested in the cold and washed in a buffer containing 20 mM Tris-HCl, pH 7.4, 0.4 M NaCl, 10% glycerol and 10 mM β-mercaptoethanol (buffer A). The cells were disrupted by three successive passages through a French press and the suspension was centrifuged at 200,000× g, 4 °C for 45 min. The pellet of membranes was washed with buffer A. The membranes were resuspended in buffer A supplemented by 2% DDM detergent. This mixture was incubated for 2 h at 4 °C under agitation before being centrifuged as described above. The supernatant containing solubilised membrane proteins was used for the purification of His_6_-BacA on Ni^2+^-NTA-agarose. The supernatant was incubated overnight at 4 °C under agitation with 2 ml of Ni^2+^-NTA-agarose polymer pre-equilibrated with buffer A supplemented with 10 mM imidazole and 0.2% DDM. The mixture was then transferred into a Poly-prep chromatography column (Biorad) and the polymer was washed with 50 ml of buffer A containing 0.2% DDM and successively 10 and 30 mM imidazole. The elution of BacA was then performed with buffer A containing 0.2% DDM and 400 mM imidazole. Fractions containing the BacA protein were subjected to an additional gel-filtration purification step on Superdex 200 (HiLoadTM 16/600 SuperdexTM 200) equilibrated in 0.1% DDM-containing buffer A. 1-ml fractions were collected and analysed by SDS-PAGE. Pure fractions were pooled and concentrated to 1 mg/ml (50000 MWCO concentrator, Vivaspin™ 20). The BacA protein concentration was estimated by measuring the absorbance at 280 nm, based on a theoretical molar extinction coefficient of 25 565 M^−1^ cm^−1^. PgpB was purified as previously described [35].

### 2.8 Synthesis of radiolabelled C_55_-PP substrate

[^14^C]C_55_-PP was synthesized using purified *E. coli* UppS synthase as previously described [8]. The reaction mixture (300 μL) containing 100 mM Hepes buffer, pH 7.5, 100 μM C_15_-PP, 800 μM [^14^C]C_5_-PP (56 kBq), 0.5 mM MgCl_2_, 50 mM KCl, 0.1% Triton X-100, and 3 μg UppS was incubated at 25 °C for 2 h. The progress of the reaction was controlled by thin layer chromatography (TLC) analysis with a 5-µL aliquot of the reaction mixture. The radiolabelled substrate (C_5_-PP) and product (C_55_-PP) were separated on pre-coated plates of silica gel 60 (Merck) using propanol/ammonium hydroxide/water (6:3:1, v/v/v) as a mobile phase and the radioactive spots were located and quantified with a radioactivity scanner (Rita Star, Raytest Isotopenmeβgeräte GmbH, Staubenhardt, Germany). [^14^C]C_55_-PP was then extracted from the reaction mixture by addition of 1 equivalent volume of 1 M pyridinium acetate, pH 4.5, and 2 volumes of butanol. It was recovered in the butanol-containing upper phase, which was stored at −20 °C. The concentration of [^14^C]C_55_-PP within the solution was measured by liquid scintillation counting of radioactivity based on the specific activity of the molecule being 1.22×10^14^ dpm/mol.

### 2.9 C_55_-PP phosphatase assay

The C_55_-PP phosphatase activity of BacA was assessed by measuring the percentage of transformation of [^14^C]C_55_-PP into [^14^C]C_55_-P upon incubation with BacA by TLC analysis. The butanol from the [^14^C]C_55_-PP solution was dried under vacuum so as to evaporate the organic solvent and to recover C_55_-PP as a lipidic film. The C_55_-PP substrate was then solubilised, at a final concentration of 50 µM, in 7 µL of 20 mM Tris-HCl, pH 7.5, 150 mM NaCl, 0.1% DDM, 1 mM CaCl_2_. The compounds (1 µL) were added to the reaction mixture at 100 µM final concentration (at the first instance) from a 2 mM stock solution prepared in DMSO. A blank experiment without compound, but with 1 µL DMSO, was performed in order to determine the 100 % of BacA activity to which all other experiments were compared. BacA (2 µL) was then added at a final concentration of 5 nM from a dilution solution prepared in the reaction buffer. The concentration of enzyme was fixed so that the consumption of substrate at the end of the incubation time in the control was less than 30%. The reaction mixture was incubated for 10 min at 37 °C and the catalysis was stopped by freezing in liquid nitrogen. The radiolabelled substrate (C_55_-PP) and product (C_55_-P) were separated by TLC on pre-coated plates of silica gel 60 (Merck) using propanol/ammonium hydroxide/water (6:3:1, v/v/v) as a mobile phase. The radioactive spots were located and quantified with a radioactivity scanner. When an inhibitory activity was observed, a range of concentrations of the corresponding inhibitor was tested to determine its IC_50_ value. Dose-response curves were plotted as BacA residual activity (RA) versus the logarithms of inhibitor concentration. Plots were analyzed by using GraphPad Prism using non-linear regression analysis and the equation used to calculate the IC_50_ values was y = 100(1 + 10^((LogIC50-x)*HillSlope))). Reported values are the average of at least three experiments. The C_55_-PP phosphatase activity of PgpB was measured as described for BacA.

### 2.10 Determination of minimum inhibitory concentration (MIC) against E. coli.

For compounds displaying inhibitory activity on BacA catalysis, their antibacterial activity was tested on different *E. coli* strains by monitoring bacterial growth in the presence of compounds (125, 250 and 500 µM). One colony was cultured in 10 ml of 2YT liquid medium and incubated at 37℃ for 6 h. The absorbance of the culture at 600 nm (A_600_) was measured to prepare a bacterial suspension in 2YT liquid medium at 15.10^6^ CFU/ml (A_600_ = 1 corresponds to 3.10^8^ CFU/ml). The bacterial suspension (200-µl) was then grown on 96-well plates in the presence or not of compounds (5-µl), which was added from a stock solution prepared in DMSO. Negative controls were then performed by growing the cells without inhibitors in 2YT medium and in 2YT medium supplemented with 2.5% of DMSO. The plates were incubated at 37℃ overnight with A_600_ measurements every 10 min using a Tecan F200 PRO microplate reader (iControl software).

### 2.11 Double-disc diffusion test

To evaluate the antibacterial activity of the compounds per se or in combination with bacitracin, against *S. aureus* RN4220 and *S. pneumoniae* R6 strains, disc diffusion method was used according to the CLSI guidelines. Bacterial suspensions equivalent to 10^8^ CFU/ml were prepared in 5 ml BHI broth. A BHI-agar plate (for *S. aureus*) or Colombia-agar plate supplemented with 5% Horse Blood (for *S. pneumoniae*) were then flooded with a bacterial suspension for 1 min to allow the bacteria to sediment before removing the excess of medium. The commercial discs loaded with 130 µg of bacitracin (Biorad) was positioned on top of the bacterial lawn at distances of 10 mm (center to center) from another sterile and blank disc, which was then loaded with 7 µL of a compound solution (50 µg). The plates were incubated at 37 °C for 24 h and the diameter of the growth inhibition zone was measured and a photo of the plates was acquired using a ChemiDoc™ MP System.

## 3 Results and discussion

### 3.1 MD simulation and virtual screening of compounds

**Fig. 4:**
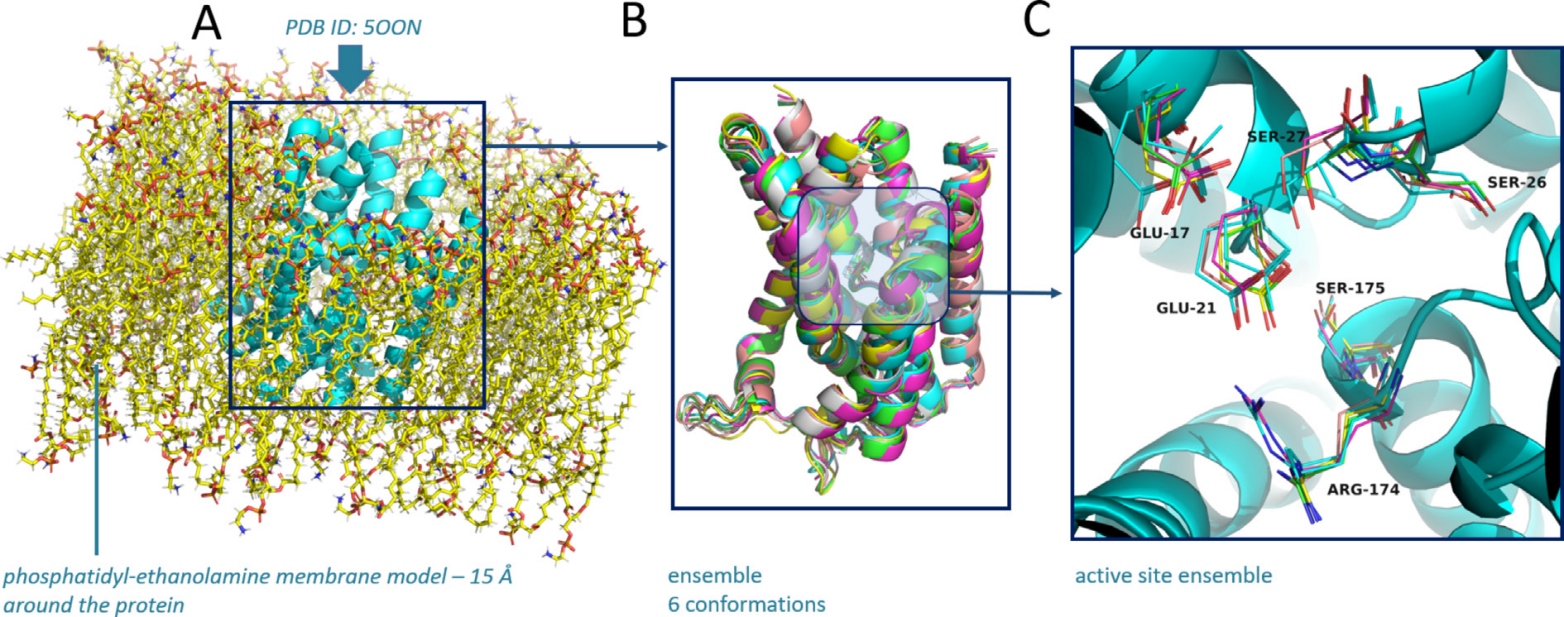
(A) Initial membrane (phosphatidyl-ethanolamine 15 Å around protein) embedded model for molecular dynamics simulation. View from the membrane plane. (B) Structural superposition of the highest-ranking clusters according to MD. (C) Conformational ensemble of the active site residues. The crystal structure is shown in a cyan cartoon model. The individual side-chain conformational ensembles of residues Glu17, Glu21, Ser27, Arg174, and Ser175 are shown as lines. View from the top of the protein into the cavity. (For interpretation of the references to colour in this figure legend, the reader is referred to the web version of this article.)

MD simulations were performed in an aqueous solvent (with a physiological concentration of 0.9% NaCl) placing the structure of BacA (PDB ID: 5OON) at 2.6 Å resolution in a 100% phosphatidylethanolamine-containing model membrane to investigate the immediate conformational flexibility of the protein and to prepare the input for ensemble docking experiments (inclusion of protein flexibility in molecular docking). No major conformational changes or domain movements were observed during MD, and the energetics and RMSD values of the protein backbone were very stable. RMSD values after alignment of 5 conformations with the initial structure ranged from 1.179 to 1.422 Å, focusing on the flexibility of the active site containing the catalytic residues Glu21, Ser27, and Arg174, which have analogous conformations in all snapshots (Fig. 4).

Nevertheless, the most distinct 5 protein conformation clusters (according to the RMSD average of pairwise linkage) were identified and the cluster centroid structures were selected for the ensemble HTVS along with the original protein crystal conformation [38]. Our goal was to identify commercially available, synthetically tractable and non-covalent inhibitors. We performed an initial screening of a prepared library of 8 million compounds using the software CCDC GOLD (GOLD 5.5, Cambridge Crystallography Data Centre, Cambridge, UK; auto-scale 0.3). We selected the 70,000 highest scoring compounds using the normalised Gold PLP scoring function to minimise the compound size bias (Fig. 3). We then performed a final exhaustive ensemble docking experiment with 70,000 compounds (auto-scale parameter of 1) and clustered the docked compounds according to structural fingerprints (hierarchical clustering, RMSD average linkage, molprint2D hashed fingerprints, Kelley criterion). For the virtual screening, we used ChemPLP scoring function that uses the ChemScore hydrogen bonding term and multiple linear potentials to model van der Waals and repulsive terms. ChemPLP is an efficient scoring function and it has been shown previously to offer superior performance in HTVS experiments compared to alternative scoring functions [32]. We employed a ChemPLP fitness score cutoff (55) for selection of compounds and covered top scoring representatives of all structural clusters to finally select a set of 100 compounds with the highest score and diversity. From the hit list, 83 compounds could be acquired from suppliers Enamine and Chembridge (the full hit list is deposited in the Supplementary Information).

### 3.2 Inhibitory activity of virtual hit compounds against BacA

The C_55_-PP substrate was synthesized by the condensation reaction of [^14^C]C_5_-PP on C_15_-PP, catalyzed by the purified UppS enzyme, as described in Material and methods section. Analysis of the reaction mixture showed a high yield of synthesis as 90% of the precursor [^14^C]C_5_-PP was converted into [^14^C]C_55_-PP (Fig. S1, panel A). After extraction of the product with butanol, a concentration of 31 µM of [^14^C]C_55_-PP was determined by measuring the radioactivity.

**Fig. 5:**
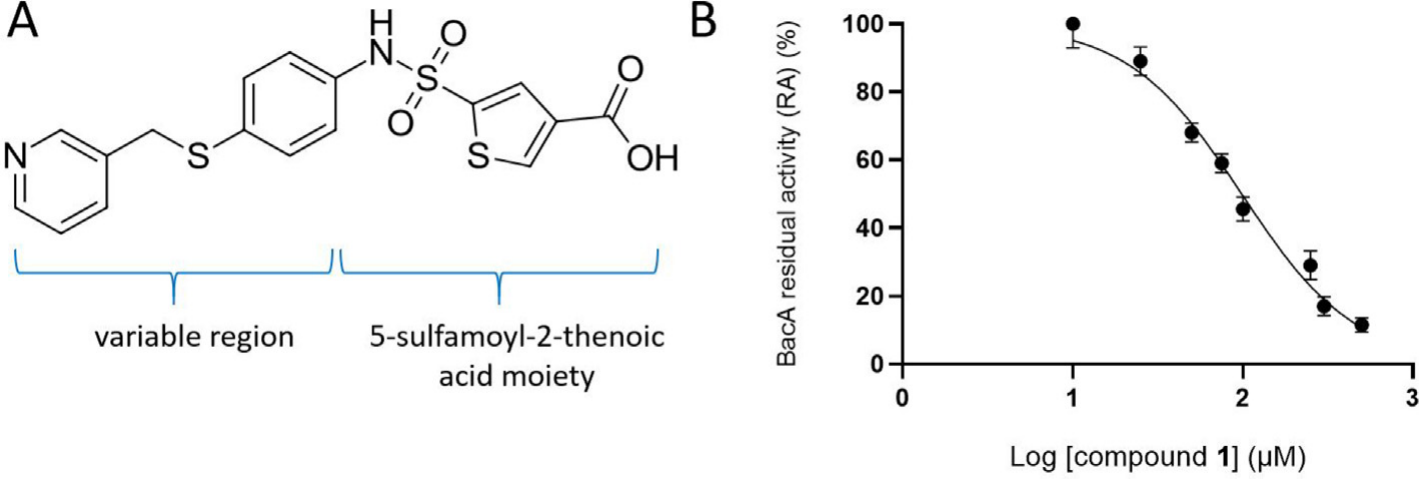
**A.** Initial hit compound **1** (5-{p-[(3-pyridyl)methylthio]phenylaminosulfonyl}-3-thenoic acid). Right-hand side of the molecule is occupied by 5-{aminosulfonyl}-3-thenoic acid moiety while the left-hand side is a “variable region”. **B.** The RA of BacA (%) plotted against the logarithm of compound **1** concentration.

The activity of BacA was then investigated by monitoring the initial velocity of [^14^C]C_55_-PP dephosphorylation, as described in Material and methods section. The inhibitory activity of the 83 hit compounds was assayed at a final concentration of 100 µM, whereas the [^14^C]C_55_-PP substrate was present at 50 µM and the BacA enzyme at 5 nM. The compounds were prepared as 2 mM DMSO stock solutions, resulting in a 5 % final concentration of DMSO in the reaction mixture. The activity of BacA without compound, but with 5 % DMSO, was defined as the reference value (100 % of C_55_-PP phosphatase activity, Fig. S1, panel B) and the activity obtained in the presence of each compound was expressed as residual activity (RA). Of the 83 virtual screening hits (Table S1), one compound, designated compound **1**, showed significant inhibition of BacA, with 45 ± 6 % RA at 100 µM. It was then assayed at a range of concentrations to determine an IC_50_ value of 98 ± 11 µM (Fig. 5 and Table 2).

**Table 2:** Collection of 7 identified hit compounds showing significant inhibition of BacA.

**Compound ID**	**MW (Da)**	**Structure**	**BacA RA (%)**^a^	**IC_50_ (µM)**	**MIC**^b^	**Off-target activity**^c^
**1**	406.50		45 ± 6	98± 11	ND	No
**2**	310.36		66± 17	132 ± 22	500 µM (155 µg/ml)	No
**3**	338.39		50 ± 14	68 ± 10	500 µM (169 µg/ml)	Yes
**4**	369.41		45 ± 10	42 ± 6	250 µM (92 µg/ml)	Yes
**5**	355.38		76 ± 13	338 ± 35	ND	No
**6**	332.37		70 ± 18	151 ± 13	250 µM (83 µg/ml)	Yes
**7**	340.39		80 ±10	366 ± 73	500 µM (170 µg/ml)	No

a RA (%) of BacA in the presence of 100 µM of compound.

b MICs were determined against BWbacA-singleΔtolC strain; ND, no growth inhibition detected up to 500 µM compound.

c Whether the compound displayed off-target antibacterial activity (Yes) or not (No), i.e. whether they have antibacterial activity against BWΔ*tolC,* is indicated.

### 3.3 Analogue identification and binding mode analysis

In order to explore the chemical space of the initial hit **1**, we searched the ZINC library for easily purchasable 5-sulfamoyl-2-thenoic acid analogues using a precalculated hit compound **1** structural similarity search at ZINC [[39], [40]]. We then selected a set of 33 new compounds that were tested against BacA (Table S2). Six new compounds **2**–**7** with significant inhibition of BacA were identified (Table 2). We confirmed the identity and determined purity of active compounds (see Supplementary Information for analytical data).

**Fig. 6:**
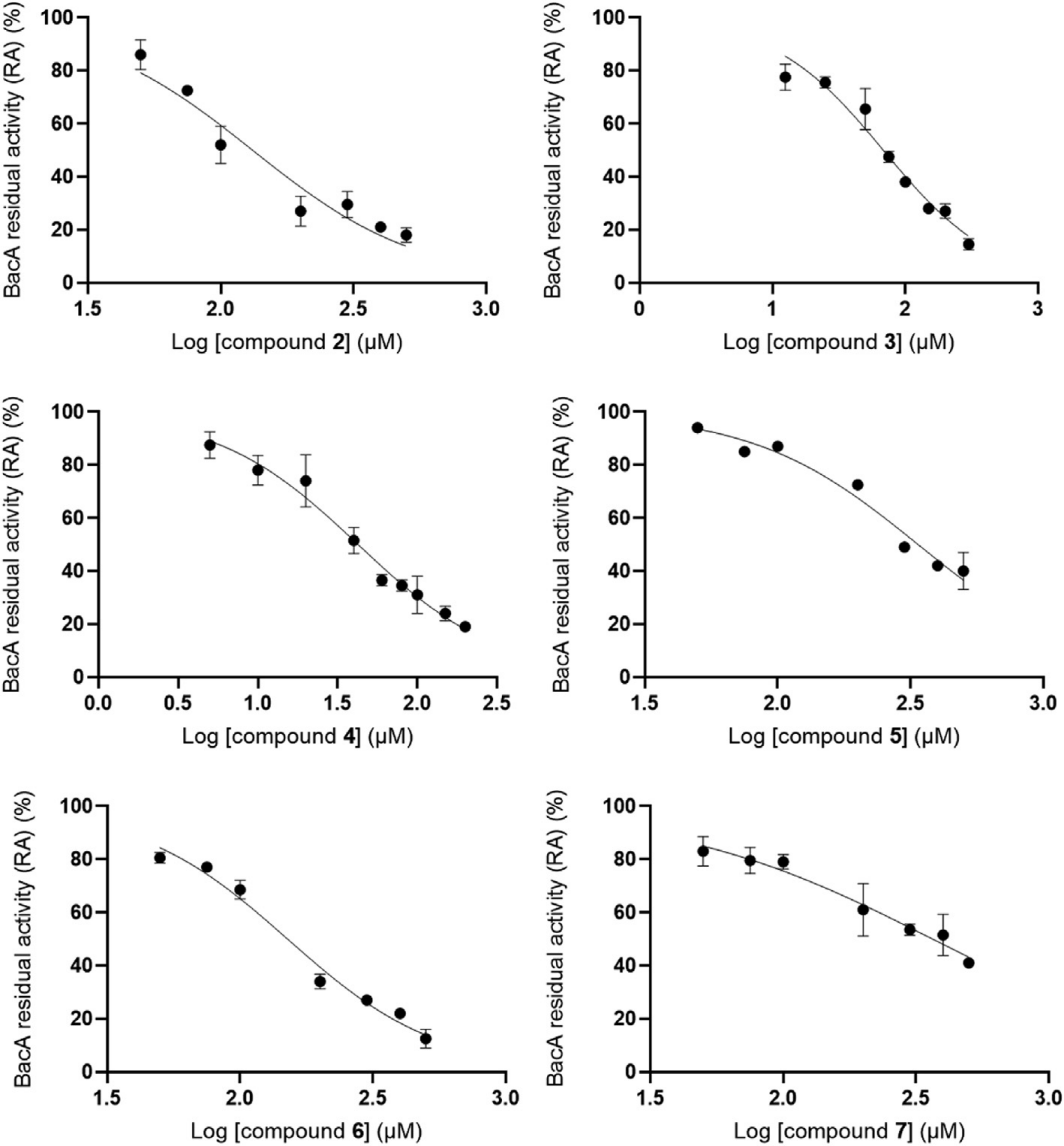
Plots of the relative activity of BacA (%) versus the logarithms of compounds **2**–**7** concentration.

The inhibitory activity of these six related compounds **2**–**7** was further assessed by varying their concentration in the reaction mixture in order to determine their IC_50_ values. The IC_50_ values of these compounds ranged from 42 µM to 366 µM (Table 2 and Fig. 6). The similarity search thus yielded two compounds (**3** and **4**) with increased potency compared to the initial hit **1**. Due to a small number of compounds, a detailed structure-activity relationship cannot be investigated. Nevertheless, a common 5-sulfamoyl-2-thenoic acid motif can be observed, with the sulfamoyl group derivatized with various aromatic or heteroaromatic systems. The variable regions (Fig. 5, panel A) of the inhibitors do not possess any functional group handles so we postulate functional group decoration and bioisosteric replacement study should be performed in the future in order to further optimize the reported 5-sulfamoyl-2-thenoic acid moiety into a viable scaffold for BacA inhibitor design. Furthermore, we are aware of the presence of the free carboxylic acid functional group and propose a bioisosteric replacement in the process of further development.

**Fig. 7:**
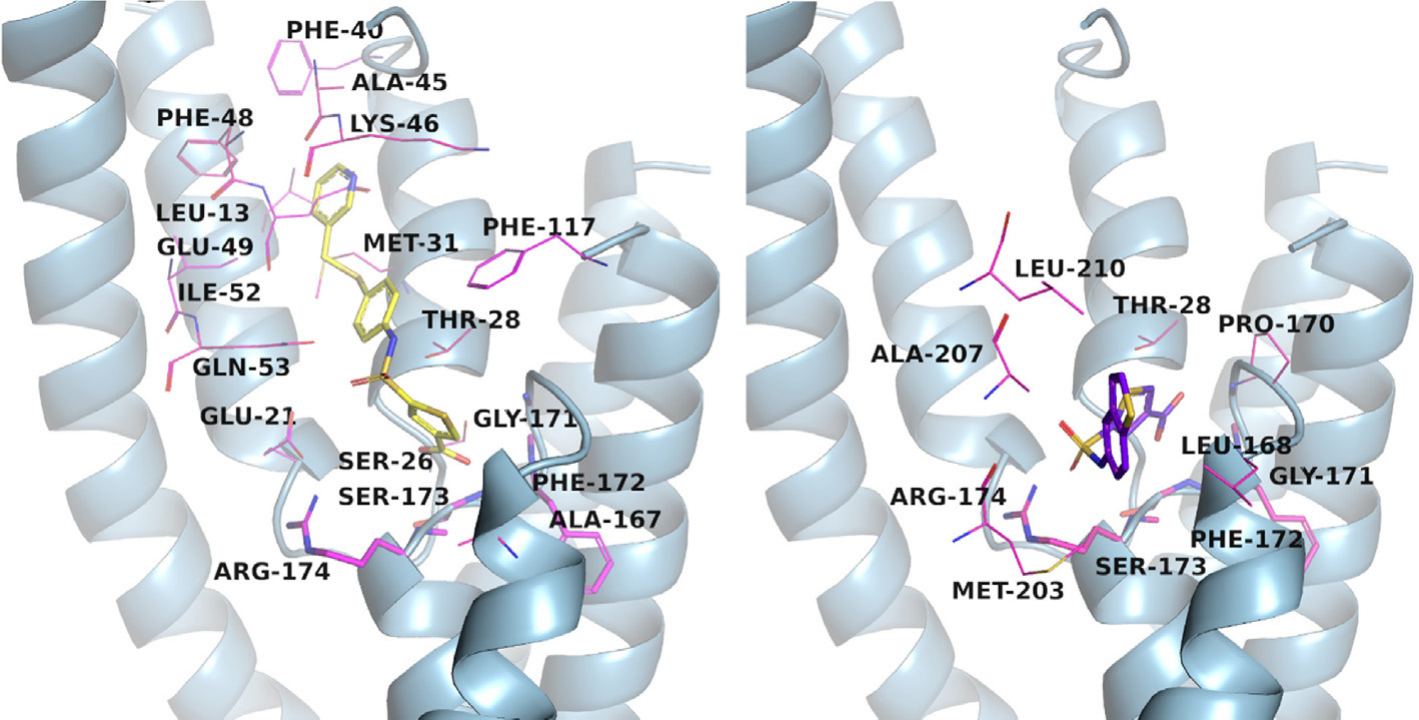
Calculated binding modes of representative hit compounds **1** in the yellow stick model (left) and **3** in the purple-blue stick model (right). The protein is shown in a light blue cartoon model, and the residues in close proximity to the hit compounds are shown in magenta line models. Residues 171–174 are shown as magenta line models as they form the bottom of the catalytic pocket. (For interpretation of the references to colour in this figure legend, the reader is referred to the web version of this article.)

Upon inspection of the docked pose of the first hit compound **1** at the BacA active site, an abundance of interactions are available (Fig. 7). Namely, the compound forms hydrophobic interactions with Leu13, Phe40, Glu49, Ile52 and Phe117. It forms hydrogen bonds with Glu21 (protein as the acceptor) and Ser26, Glu49, Gln53, Gly171, Phe172, Ser173, and Arg174, all with protein as the donor. Furthermore, cation-π interaction with Arg174 can be observed. The common 5-sulfamoyl-2-thenoic acid moiety is thus found at the catalytic polar pocket defined by Gly171, Phe172, Ser173 and Arg174 while the tail is located at the more hydrophobic binding site entrance. A second binding mode can be observed for smaller hit compounds and upon inspection of compound **3**, similar hydrogen bonding interactions are present between the 5-sulfamoyl-2-thenoic acid moiety and Gly171, Ser173 together with key catalytic Arg174, all with protein as the donor (Fig. 7). The aromatic distal end is positioned laterally to the binding site entrance, making hydrophobic interactions with Leu168 and Ala207. Other hit compounds behave analogously and the calculated binding poses also converged in both cases of ionised and protonated sulfamoyl moieties firmly placing 5-sulfamoyl-2-thenoic acid in the catalytic binding pocket near Arg174.

**Fig. 8:**
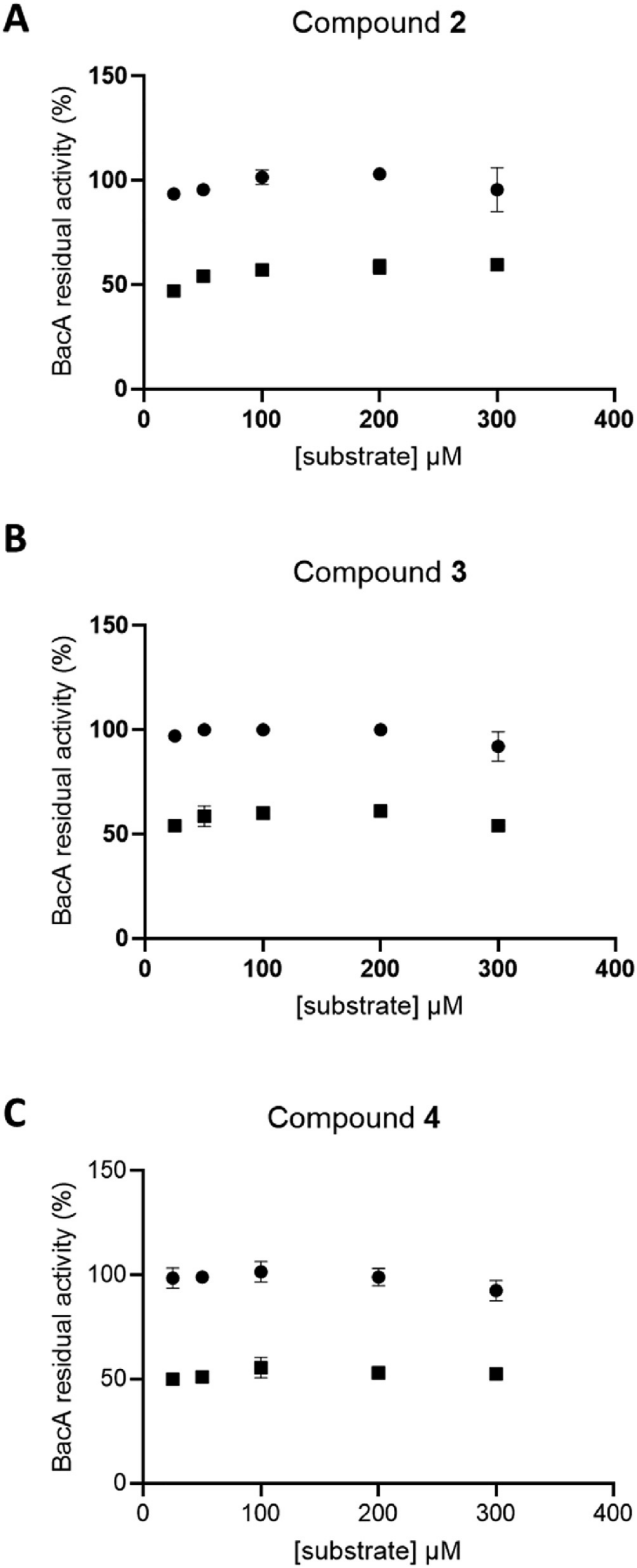
Compound versus substrate competition kinetic experiments. The residual activity of BacA was measured at various concentrations of substrate in the absence (circle) and presence (square) of 200 µM compound 2 (A), 3 (B) and 4 (C). Plots of BacA residual activity versus the concentration of substrate.

In order to determine the nature of the inhibition event, we further performed competition kinetic experiments. We have measured the specific activity of BacA at various concentrations of substrate in the absence or in the presence of compounds **2**, **3** and **4**. Of note, we observed an important inhibition of BacA activity by an excess of substrate above 300 µM (data not shown), preventing us to perform a full enzyme kinetic experiment. Nevertheless, we performed kinetic analysis within the frame of 25 to 300 µM of substrate in the presence and the absence of 200 µM of compounds. As shown in Fig. 8, BacA enzyme was apparently saturated in this frame work since the activity does not vary. Then, we observed the same inhibition rate in the presence of compound whichever the substrate concentration. Therefore, the data are strongly suggestive that these compounds are non-competitive inhibitors. This can be expected as the apo-form structural information used in the structure-based drug design does not provide insight into native substrate positioning at the active site and the complete volume around active site key residues was used for molecular docking experiment.

### 3.4 Antibacterial activity of BacA inhibitors

**Fig. 9:**
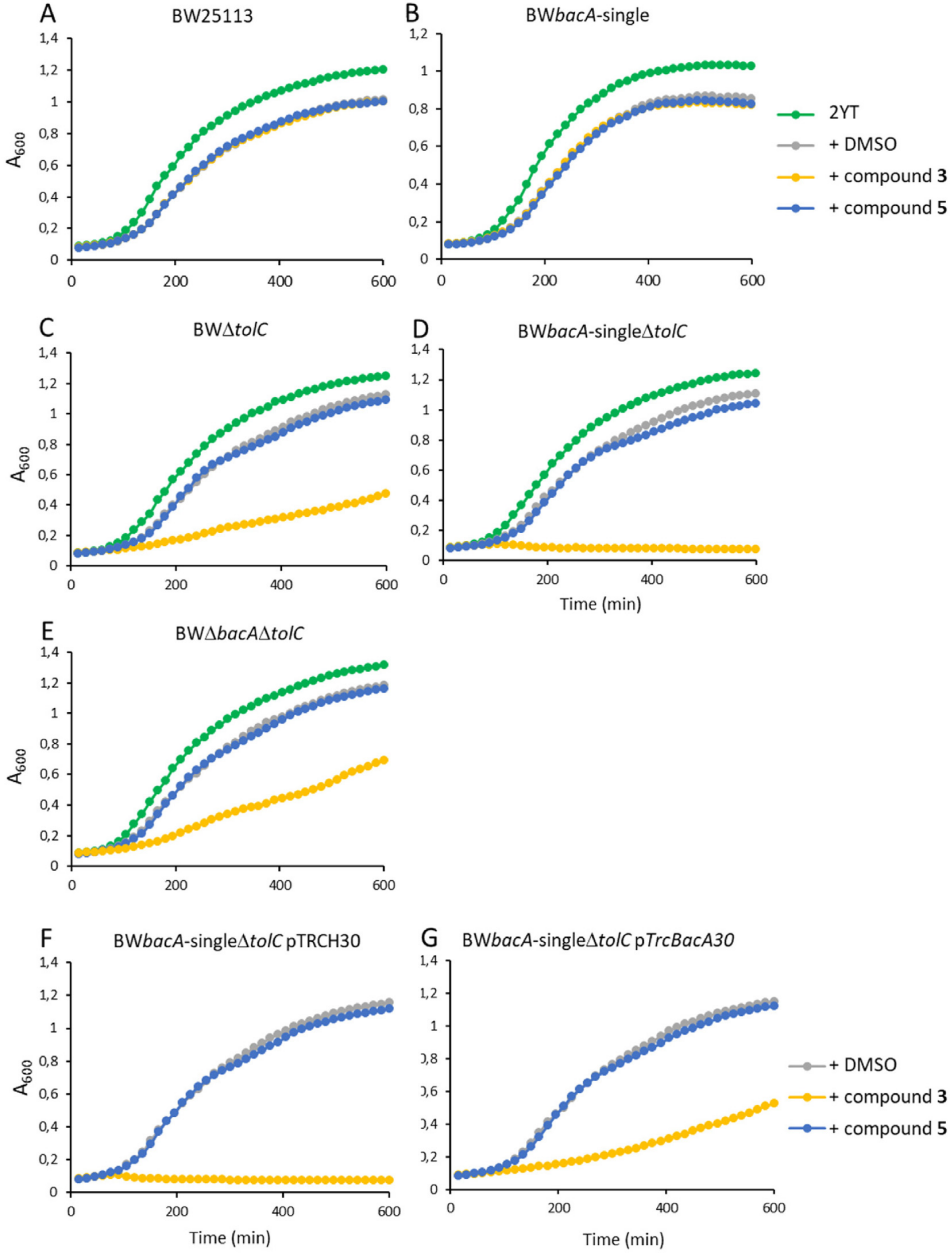
Antibacterial activity evaluation of compounds **3** and **5** against different BW25113 *E. coli* variants. Each panel corresponds to one variant strain, whose name (genotype) is indicated on top. While compound **5** did not display any antibacterial activity, compound **3** caused growth inhibition upon inactivation of TolC and/or the PAP2 C_55_-PP phosphatases (i.e. *pgpB*, *ybjG* and *lpxT* deletions). A complete growth inhibition was observed with BW*bacA*-singleΔ*tolC* strain, while solely a moderate inhibition was observed with BWΔ*tolC* and BWΔ*bacA*Δ*tolC* strains, thus strongly suggesting that the arrest of growth arises through BacA inhibition but also that the compound displays off-target activity. The relative specificity of compound **3** towards BacA was confirmed by showing that in trans overexpression of *bacA* in BW*bacA*-singleΔ*tolC* strain partially alleviated growth inhibition (we restored similar growth as for BWΔ*bacA*Δ*tolC* strain). Representative growth curves of bacterial cells obtained in 2YT medium in the absence or the presence of compound **3** and **5** at 500 µM.

Two compounds, **3** and **5**, were assessed for antibacterial activity using the broth microdilution method against the *E. coli* BW25113 wild type strain. No growth inhibition of these cells was observed in the presence of compounds (Fig. 9, panel A). Considering the plurality of C_55_-PP phosphatases in *E. coli*, the inhibition of BacA may not induce growth inhibition as long as the PAP2 enzymes (PgpB, YbjG, and LpxT) are functional. Consequently, compounds **3** and **5** were tested against a BW25113 variant in which the PAP2 enzymes were depleted (DMEG9 strain, alias BW*bacA*-single). In this case, C_55_-P recycling relies only on BacA. Nevertheless, again no growth inhibition was observed (Fig. 9, panel B).

*E. coli* cells express an efflux pump, consisting of AcrA, AcrB and TolC proteins, which expels various noxious compounds from the cytoplasm, plasma membrane, and periplasm out of the cell. Consequently, the compounds might be expelled before they can bind BacA. Accordingly, the compounds were tested against a BW25113 variant depleted of TolC (BWΔ*tolC*). In this case, compound **3** exhibited significant growth inhibition, by increasing the doubling time of cells by 3-fold (Fig. 9, panel C), thereby indicating that i) inactivation of TolC was required for this compound to exhibit antibacterial activity, and ii) compound **3** is recognized as a substrate by the pump. In contrast, compound **5** did not lead to any growth inhibition of this strain.

Next, compounds **3** and **5** were tested against a strain combining the depletions of PAP2 enzymes together with TolC (BW*bacA*-singleΔ*tolC*). In this case, a total growth inhibition was observed with 500 µM of compound **3**, while no growth inhibition occurred with compound **5** (Fig. 9, panel D). These data demonstrate a synergistic effect of combining PAP2 and TolC depletions for the antibacterial activity of compound **3**. The fact that the depletion of the PAP2 enzymes strongly enhances the effect of this compound on bacterial growth suggests that the observed antibacterial activity implies BacA inhibition. Nevertheless, to determine whether compound **3** also exhibits any BacA-independent antibacterial activity, this compound was also tested against BW25113 variant, depleted of both BacA and TolC (but possessing the PAP2 enzymes). Compound **3** exhibited similar antibacterial activity whether BacA was depleted or not, in a TolC depletion background, demonstrating that the compound displays a significant, although moderate, BacA-independent activity (Fig. 9, panel E).

To further assess the specificity of compounds **3** and **5** with respect to BacA, they were tested against the strain depleted of both TolC and PAP2 but overexpressing an ectopic copy of the *bacA* gene on a plasmid. As expected, compound **3** caused total growth inhibition when used at 500 µM against the control strain carrying the empty vector (pTRCH30) (Fig. 9, panel F). Upon *bacA* overexpression, the susceptibility was decreased at the level observed with the strain depleted of TolC alone (Fig. 9, panel G). These data show that the overexpression of BacA alleviates the antibacterial activity of compound **3**, in the same way as having functional PAP2 enzymes. They confirm that compound **3** interferes with BacA, causing total arrest of growth unless BacA is overexpressed to withstand the inhibitory activity of the compound. Nevertheless, when BacA was no longer present, a residual susceptibility to compound **3** was observed due to another unknown off-target effect of the molecule (Fig. 9, panel E).

In order to determine MIC values and to assess the specificity of action towards BacA, all compounds displaying BacA inhibition were also tested against the TolC and TolC/PAP2-depleted strains (Fig. S2 and Table 2). Compounds **1** and **5** showed no growth inhibition up to 500 µM. Compounds **2** and **7** displayed total growth inhibition of TolC/PAP2-depleted strains (MIC = 500 µM) and had no effect on TolC-depleted strain, suggesting that these compounds have less off-target effect as compared to compound **3**; i.e. they exhibit more specific activity towards BacA. Among the other inhibitors, compounds **4** and **6** displayed the lowest MIC value, 250 µM on TolC/PAP2-depleted strain, with no effect on TolC-depleted strain; i.e. they have higher antibacterial efficacy directed against BacA and minor off-target effect at this concentration. At 500 µM, the latter compounds displayed the same effect as compound **3**, i.e., total growth inhibition against TolC/PAP2-depleted strain and a moderate inhibition of TolC-depleted strain.

In order to determine whether the PAP2 C_55_-PP phosphatases could be the off-targets of our hit compounds, which could account for the growth inhibition of BWΔ*tolC* strain, we tested the potential inhibitory activity of compounds **3** and **4** against purified PgpB enzyme. PgpB was not inhibited in the presence of 100 µM of the latter compounds (data not shown) suggesting that off-targets are not linked to the recycling process of C_55_-PP and could be any other essential cellular activity.

As already mentioned, it was reported that the deletion of *bacA* gene in *S. aureus* and *S. pneumoniae* abolishes the virulence of these pathogenic bacteria, but it also increases their susceptibility to bacitracin by 16- and more than 1000-fold, respectively [15]. Therefore, we tested whether BacA inhibitors increase the bacterial susceptibility to bacitracin of both species by using double-disc diffusion tests. As shown on Fig. S3, panel A, none of the tested compounds were found to display any growth inhibition, nor to increase the bacitracin activity, towards *S. aureus*. In contrast, against *S. pneumonia*, compound **4**, which exhibited the lowest IC_50_ and MIC against BacA and *E. coli* cells, respectively, increased bacitracin susceptibility (Fig. S3, panel B). Indeed, while the compound **4** didn’t display antibacterial activity per se (i.e. no inhibition zone was observed around compound 4-loaded disc), its proximity with bacitracin significantly increased the inhibition zone of the latter antibiotic (from 7.5 mm to 10 mm). The other compounds didn’t show any activity per se or in combination with bacitracin towards *S. pneumonia*. These data strongly suggest that our best hit compound towards BacA from *E. coli*, is able to target BacA from distant species in vivo.

## 4 Conclusion

In this work, we performed a robust and comprehensive high-throughput virtual screening using the available crystal structure of the undecaprenyl pyrophosphate phosphatase BacA from *E. coli*. We used a prepared library of 8 million unique compounds to identify new commercially available, synthetically tractable non-covalent inhibitors of BacA. Using ensemble docking and compound scaffold analysis, we acquired and biochemically evaluated a total of 116 compounds to find seven small molecule BacA inhibitors with IC_50_ values ranging from 42 to 366 µM. All active compounds have a 5-sulfamoyl-2-thenoic acid moiety on one side and variable aromatic systems on the other side of the molecule. We hypothesise that the variable aromatic region should be a good starting point for the design and optimization of future compounds. Indeed, biological evaluation has shown that compound **4** has an IC_50_ of 42 ± 6 µM for BacA and an MIC of 92 µg/ml against TolC/PAP2-depleted strains, suggesting that it is able to target protein in bacteria but is also a substrate for the efflux pump. We present a series of valuable experimentally identified inactive compounds that can serve as experimental decoys for future computational discovery of BacA inhibitors and, more importantly, seven initial BacA inhibitors that provide a solid foundation for future development of BacA-targeted antibacterial agents.

## Declaration of Competing Interest

The authors declare that they have no known competing financial interests or personal relationships that could have appeared to influence the work reported in this paper.
