# Supplementary material for: Towards discovery of inhibitors of the undecaprenyl-pyrophosphate phosphatase BacA by virtual high-throughput screening
Source: Comput Struct Biotechnol J. 2022 May 11;20:2360–71. doi: 10.1016/j.csbj.2022.05.010 (PMC9127117; doi:10.1016/j.csbj.2022.05.010)
Supplement: Supplementary data 1 [file mmc1.pdf]

Supplementary Information

for

Towards discovery of inhibitors of the undecaprenyl-pyrophosphate phosphatase BacA by virtual high-throughput screening

Marko Jukič [1, 2, a], Rodolphe Auger [3, a], Victor Folcher [3], Matic Proj [4], Hélène Barreteau [3], Stanislav Gobec [4 *] and Thierry Touzé [3 *]

[1] – University of Maribor, Faculty of Chemistry and Chemical Engineering, Laboratory of Physical Chemistry and Chemical Thermodynamics, Smetanova 17, SI-2000 Maribor, Slovenia.

[2] – University of Primorska, Faculty of Mathematics, Natural Sciences and Information Technologies, Glagoljaška 8, SI-6000 Koper, Slovenia.

[3] – Université Paris-Saclay, CEA, CNRS, Institute for Integrative Biology of the Cell (I2BC), FR-91198, Gif-sur-Yvette, France

[4] – Univerza v Ljubljani, Fakulteta za Farmacijo, Aškerčeva cesta 7, SI-1000 Ljubljana, Slovenia

[*] - Correspondence: Thierry Touzé, Université Paris-Saclay, CEA, CNRS, Institute for Integrative Biology of the Cell (I2BC), FR-91198, Gif-sur-Yvette, France, e-mail: thierry.touze@universite-paris-saclay.fr; Stanislav Gobec, Univerza v Ljubljani, Fakulteta za Farmacijo, Aškerčeva cesta 7, SI-1000 Ljubljana, Slovenia, e-mail: stanislav.gobec@ffa.uni-lj.si.

[a] These authors have contributed equally to this work and share first authorship.

Complete Compound List

Table S1: Initial hitlist 83 compound purchase

No.	Compound	Mr (g/mol)	formula
1	UL-FFA_BAC-1	587.2	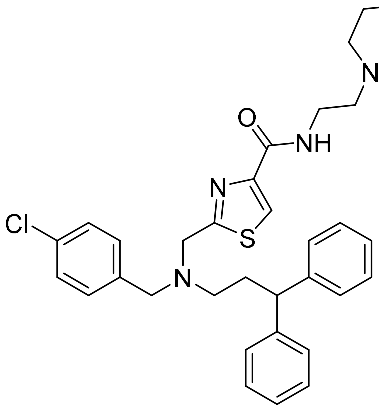
2	UL-FFA_BAC-2	573.78	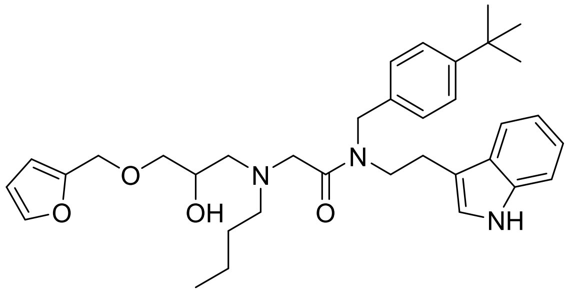
3	UL-FFA_BAC-3	563.14	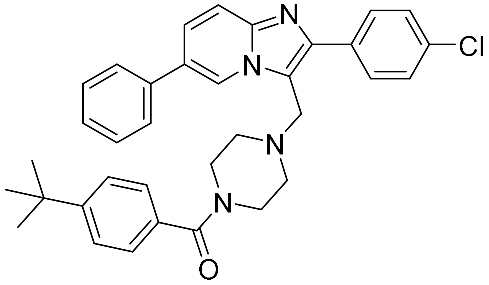
4	UL-FFA_BAC-4	495.06	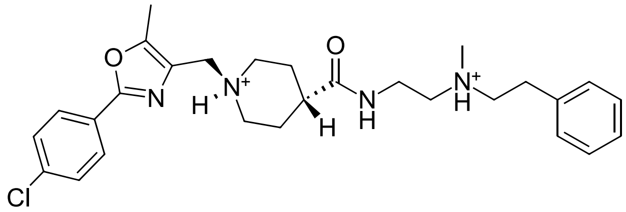
5	UL-FFA_BAC-5	497.65	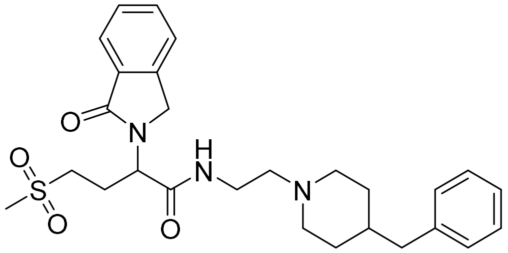

6	UL-FFA_BAC-6	404.51	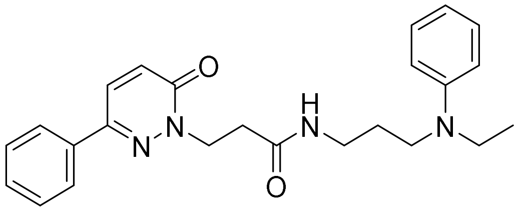
7	UL-FFA_BAC-7	517.44	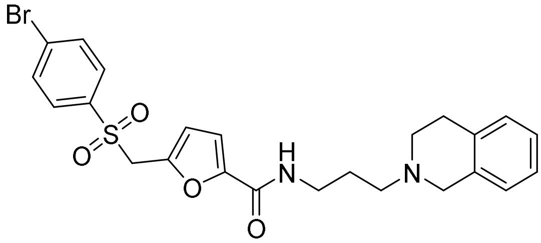
8	UL-FFA_BAC-8	457.63	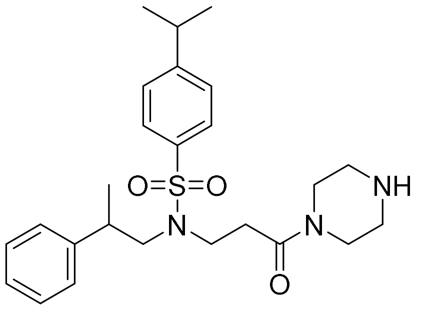
9	UL-FFA_BAC-9	626.82	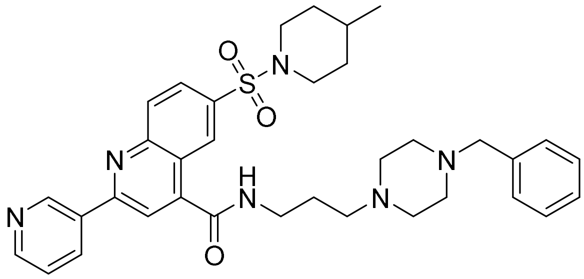
10	UL-FFA_BAC-10	387.44	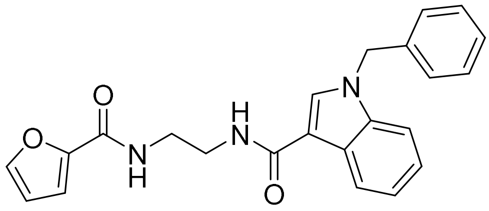
11	UL-FFA_BAC-11	402.50	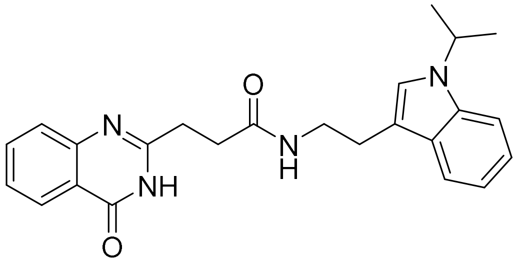
12	UL-FFA_BAC-12	376.48	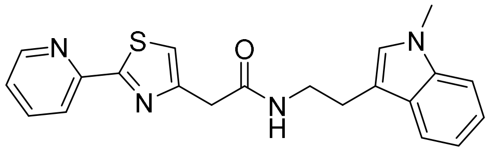
13	UL-FFA_BAC-13	372.43	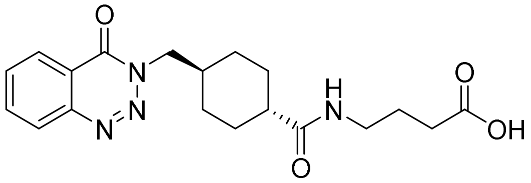

14	UL-FFA_BAC-14	318.37	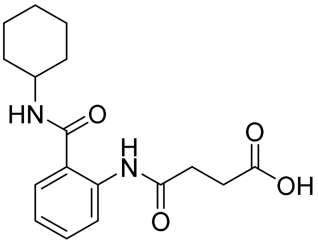
15	UL-FFA_BAC-15	427.54	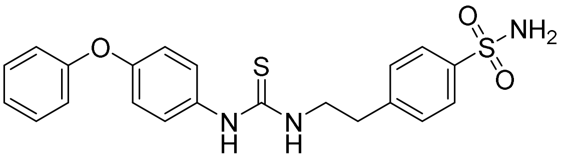
16	UL-FFA_BAC-16	369.37	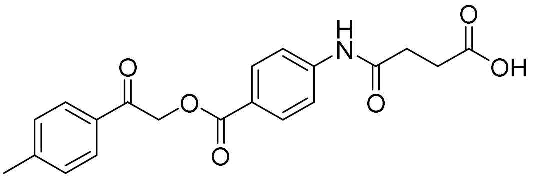
17	UL-FFA_BAC-17	398.48	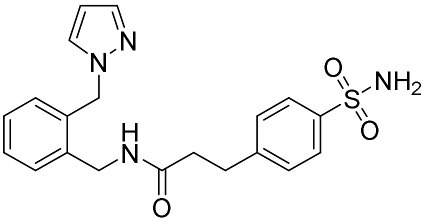
18	UL-FFA_BAC-18	432.56	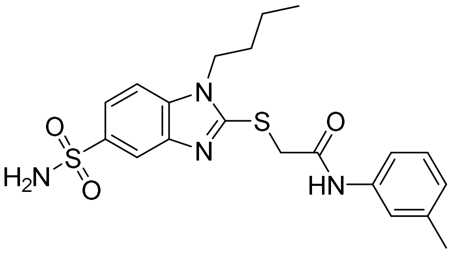
19	UL-FFA_BAC-19	400.51	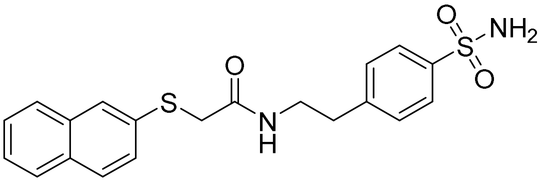
20	UL-FFA_BAC-20	490.59	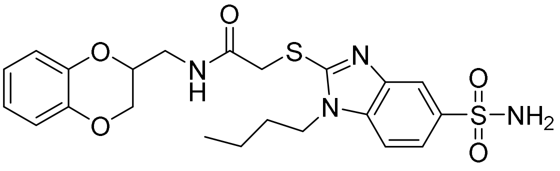
21	UL-FFA_BAC-21	420.49	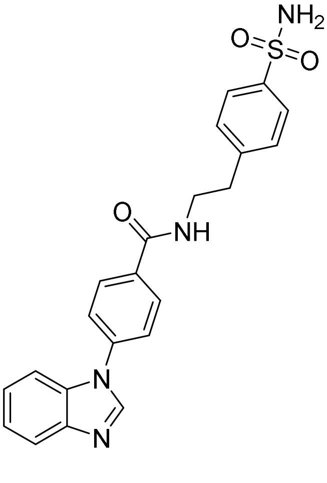

22	UL-FFA_BAC-22	395.48	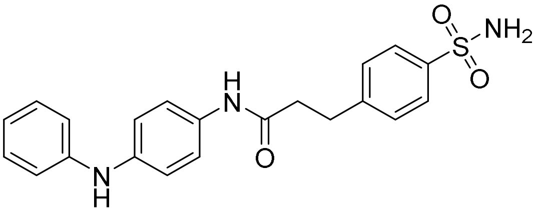
23	UL-FFA_BAC-23	384.45	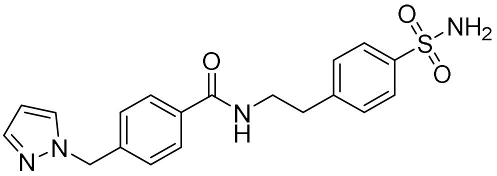
24	UL-FFA_BAC-24	404.5	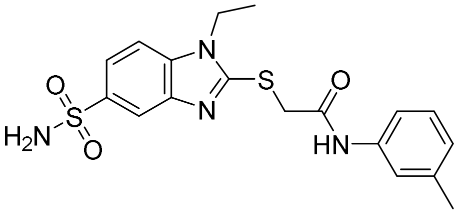
25	UL-FFA_BAC-25	309.369	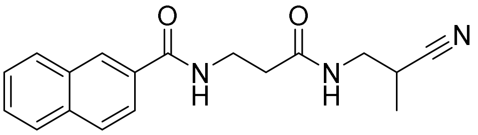
26	UL-FFA_BAC-26	319.42	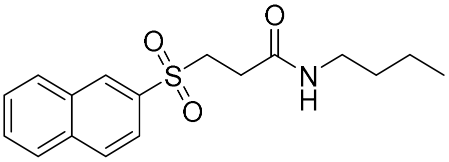
27	UL-FFA_BAC-27	420.49	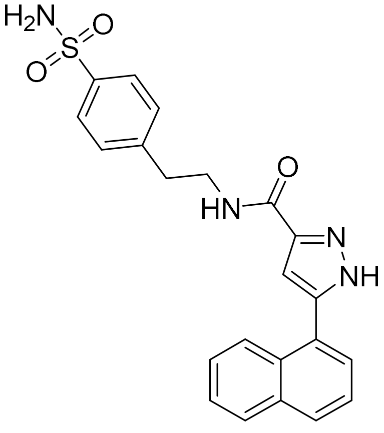
28	UL-FFA_BAC-28	401.42	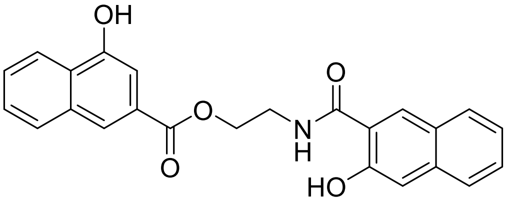
29	UL-FFA_BAC-29	399.45	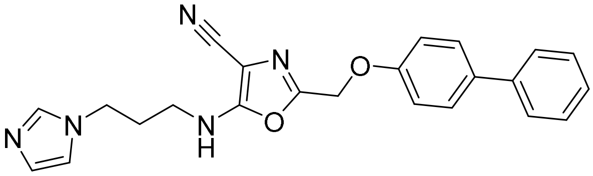

30	UL-FFA_BAC-30	457.96	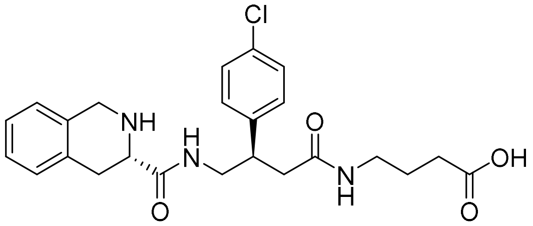
31	UL-FFA_BAC-31	535,64	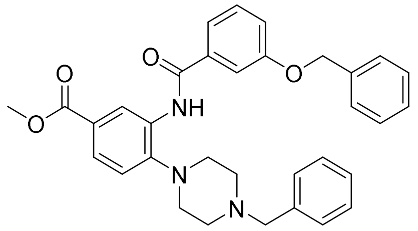
32	UL-FFA_BAC-32	*486.64*	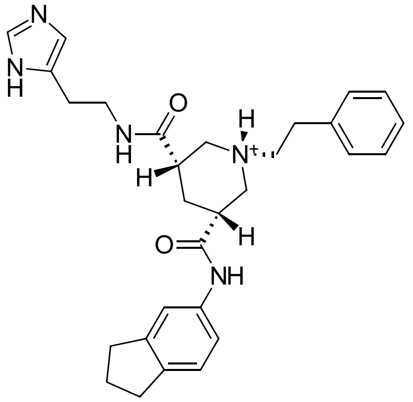
33	UL-FFA_BAC-33	*473.60*	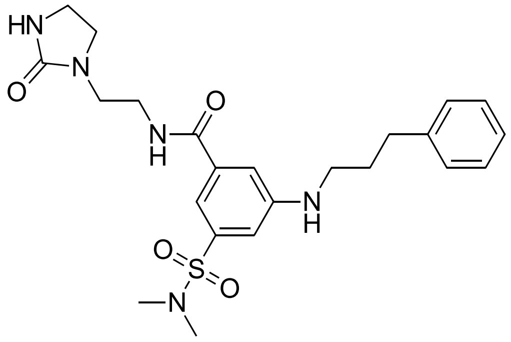
34	UL-FFA_BAC-34	*354.42*	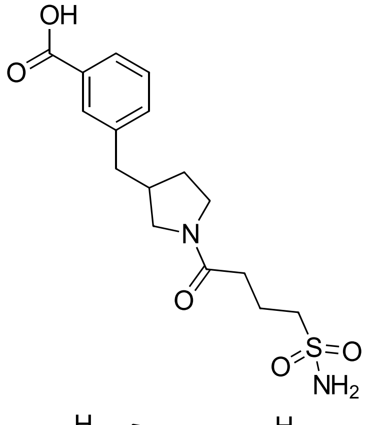
35	UL-FFA_BAC-35	*363.48*	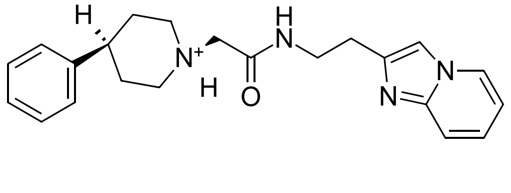

36	UL-FFA_BAC-36	*331.42*	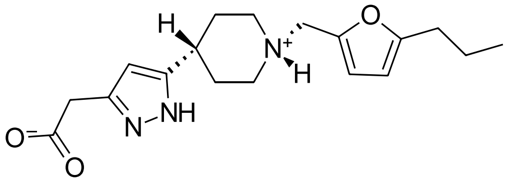
37	UL-FFA_BAC-37	330.39	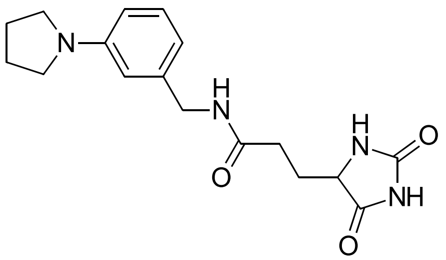
38	UL-FFA_BAC-38	*285.35*	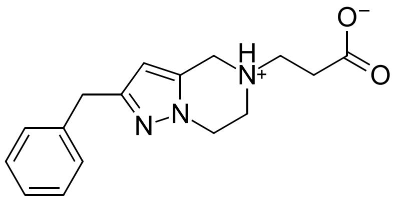
39	UL-FFA_BAC-39	*367.48*	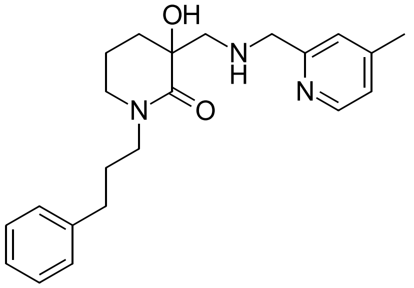
40	UL-FFA_BAC-40	*305.33*	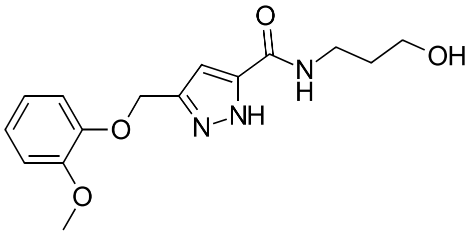
41	UL-FFA_BAC-41	*349.39*	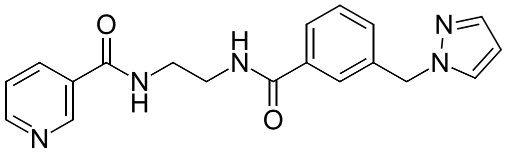
42	UL-FFA_BAC-42	*370.52*	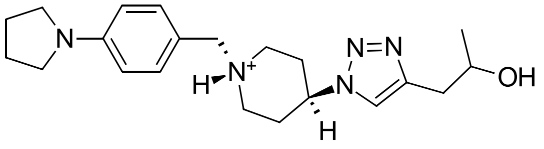
43	UL-FFA_BAC-43	*372.88*	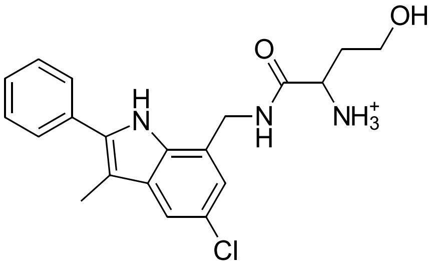

44	UL-FFA_BAC-44	*342.40*	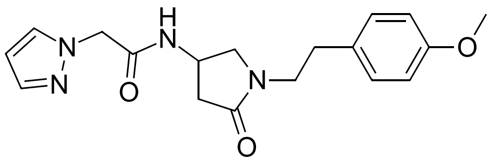
45	UL-FFA_BAC-45	*377.53*	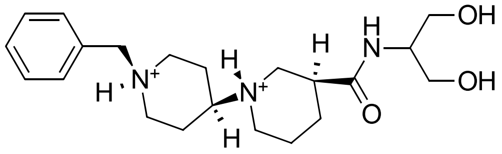
46	UL-FFA_BAC-46	405.50	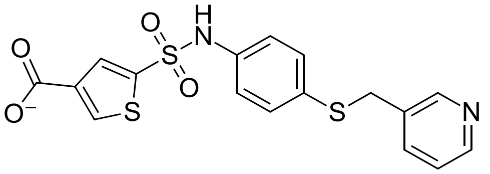
47	UL-FFA_BAC-47	335.35	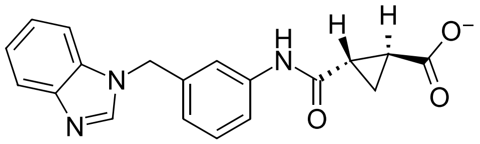
48	UL-FFA_BAC-48	335.40	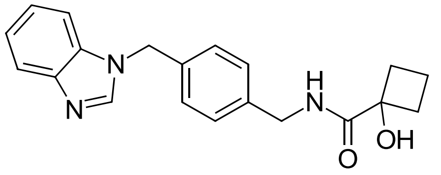
49	UL-FFA_BAC-49	304.30	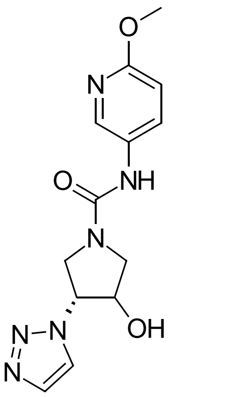
50	UL-FFA_BAC-50	314.38	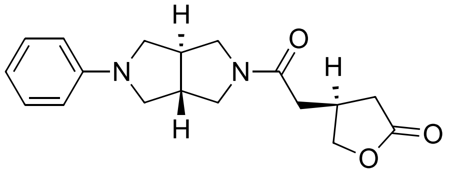
51	UL-FFA_BAC-51	334.38	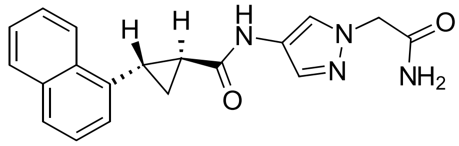
52	UL-FFA_BAC-52	320.45	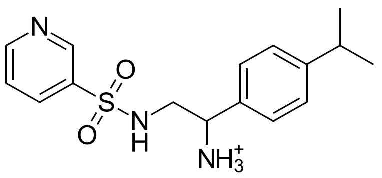

53	UL-FFA_BAC-53	368.48	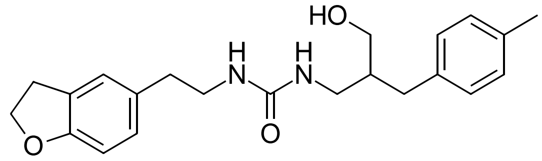
54	UL-FFA_BAC-54	290.35	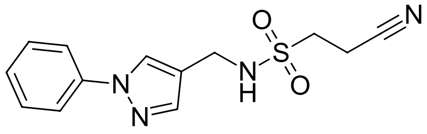
55	UL-FFA_BAC-55	356.44	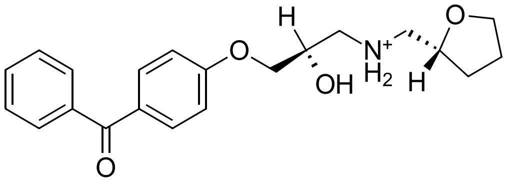
56	UL-FFA_BAC-56	442.54	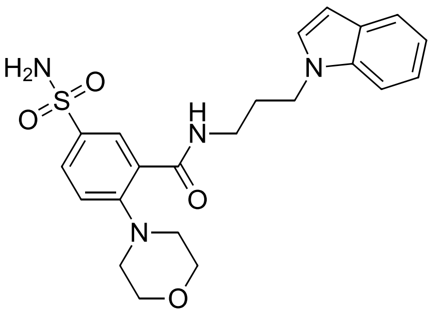
57	UL-FFA_BAC-57	301.37	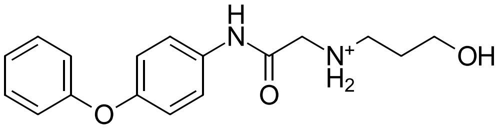
58	UL-FFA_BAC-58	456.61	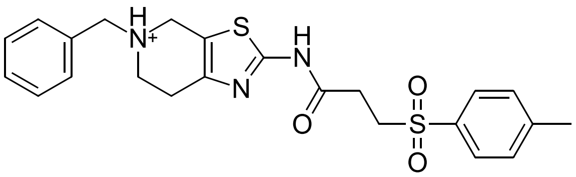
59	UL-FFA_BAC-59	330.45	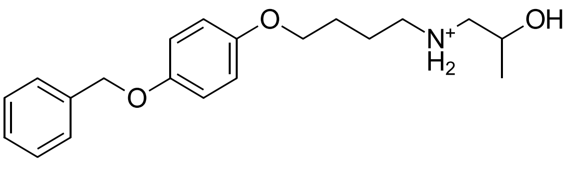
60	UL-FFA_BAC-60	458.65	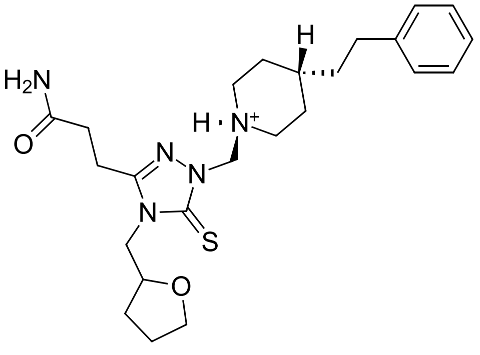
61	UL-FFA_BAC-61	340.42	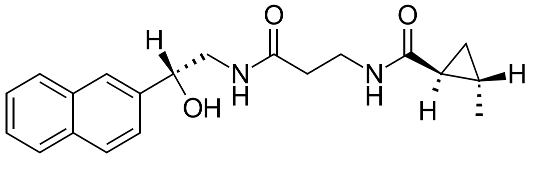

62	UL-FFA_BAC-62	303.41	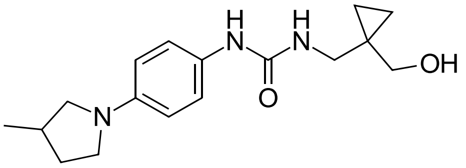

BAC-63 – BAC-86 in Table S2

63	UL-FFA_BAC-87	370.49	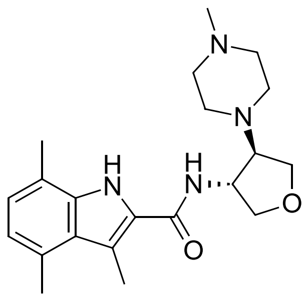
64	UL-FFA_BAC-88	533.58	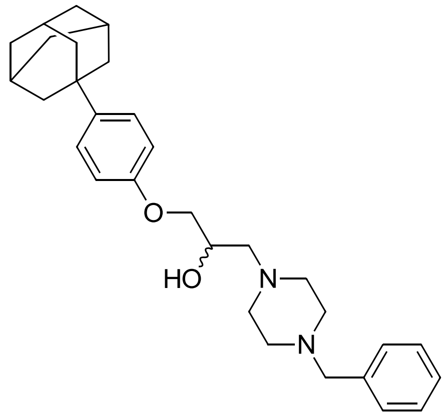
65	UL-FFA_BAC-89	369.51	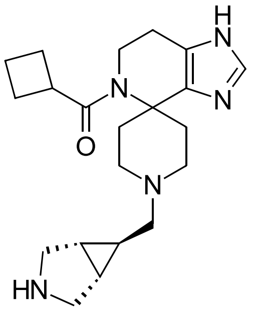
66	UL-FFA_BAC-90	373.46	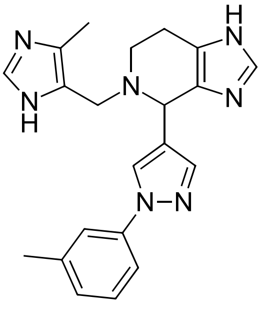
67	UL-FFA_BAC-91	399.49	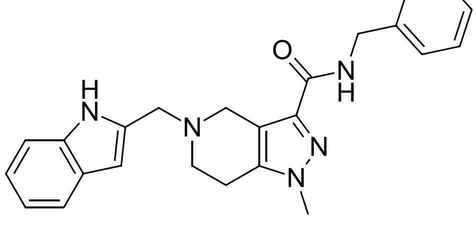

68	UL-FFA_BAC-92	400.48	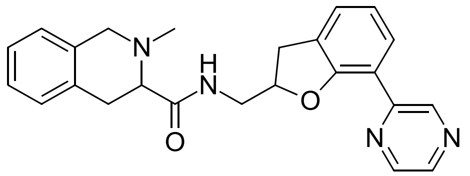
69	UL-FFA_BAC-93	389.50	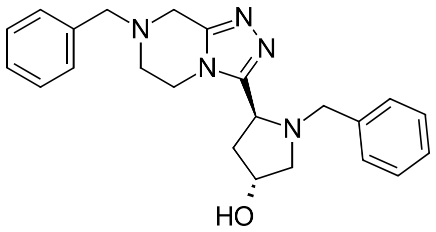
70	UL-FFA_BAC-94	424.52	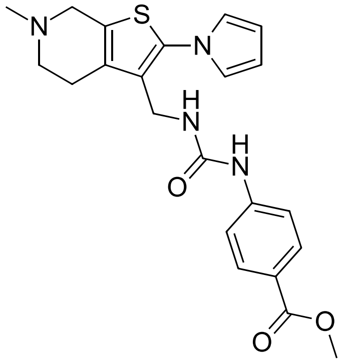
71	UL-FFA_BAC-95	534.1	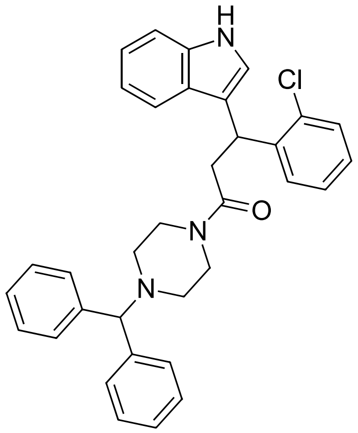
72	UL-FFA_BAC-96	408.50	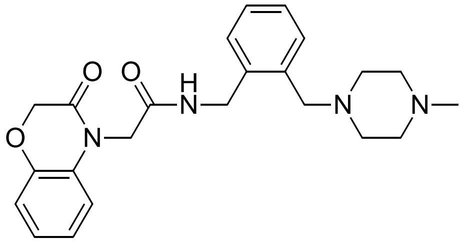
73	UL-FFA_BAC-97	412.40	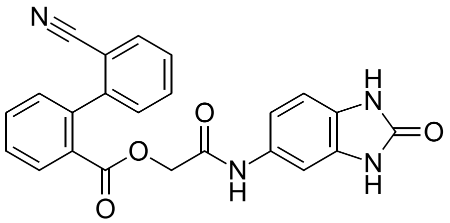
74	UL-FFA_BAC-98	348.41	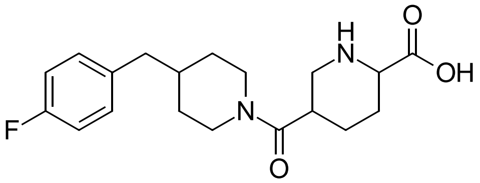

75	UL-FFA_BAC-99	377.44	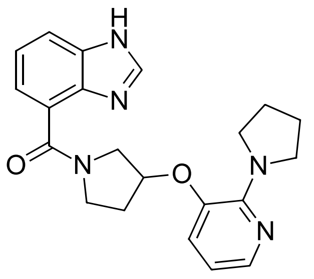
76	UL-FFA_BAC-100	430.59	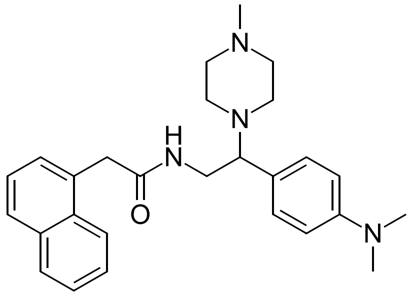
77	UL-FFA_BAC-101	421.58	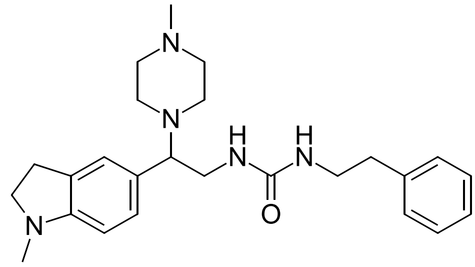
78	UL-FFA_BAC-102	502.66	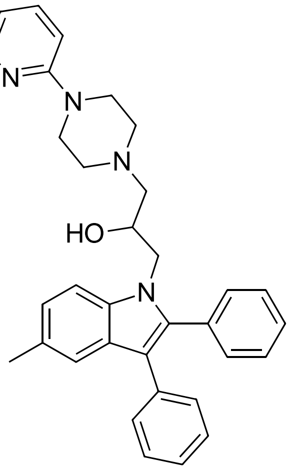
79	UL-FFA_BAC-103	467.24	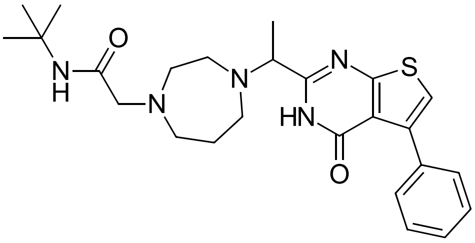

80	UL-FFA_BAC-104	546.37	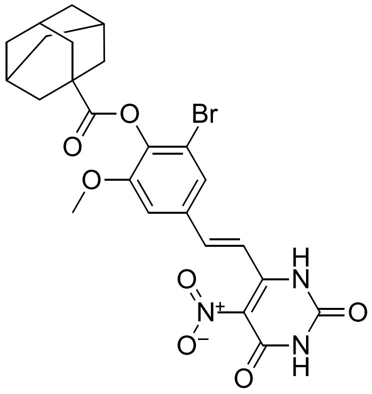
81	UL-FFA_BAC-105	462.62	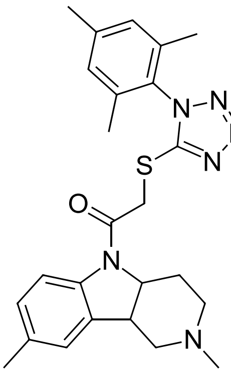
82	UL-FFA_BAC-106	469.54	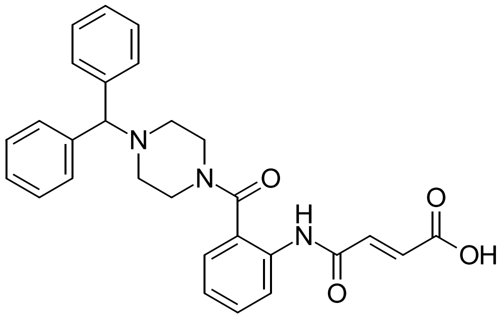
83	UL-FFA_BAC-107	351.49	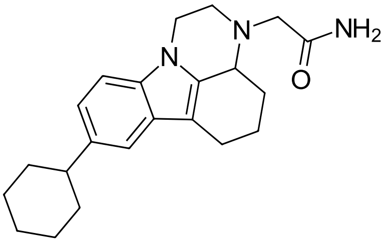

Table S2: Hit compound analogue purchase (33 compounds in total)

No.	Compound	Mr (g/mol)	formula
1	UL-FFA_BAC-63	*398.50*	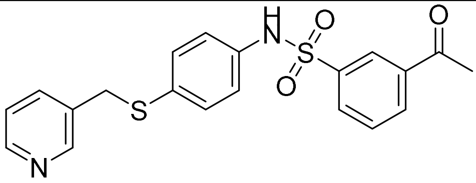
2	UL-FFA_BAC-64	*400.47*	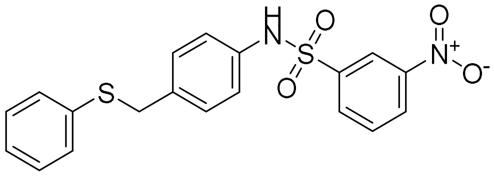

3	UL-FFA_BAC-65	*368.40*	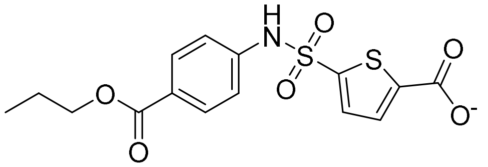
4	UL-FFA_BAC-66	*416.45*	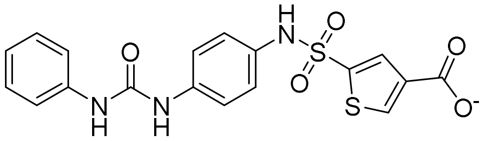
5	UL-FFA_BAC-67	*310.36*	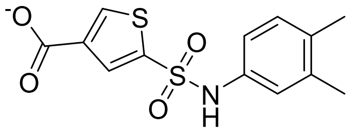
6	UL-FFA_BAC-68	*379.47*	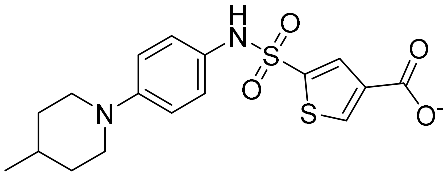
7	UL-FFA_BAC-69	*338.39*	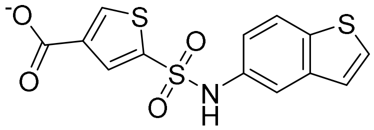
8	UL-FFA_BAC-70	*369.41*	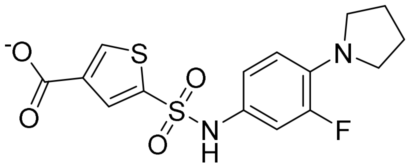
9	UL-FFA_BAC-71	*343.41*	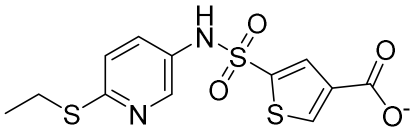
10	UL-FFA_BAC-72	*364.42*	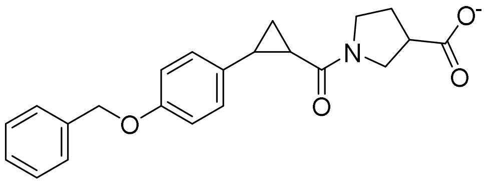
11	UL-FFA_BAC-73	*355.38*	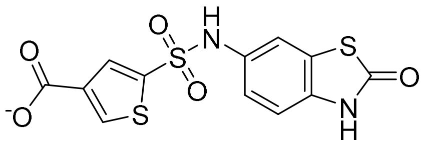
12	UL-FFA_BAC-74	*326.36*	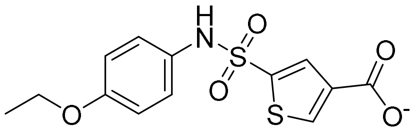
13	UL-FFA_BAC-75	*404.45*	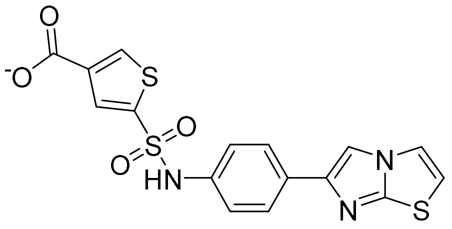
14	UL-FFA_BAC-76	*332.37*	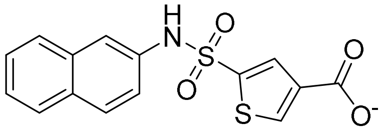

15	UL-FFA_BAC-77	*423.44*	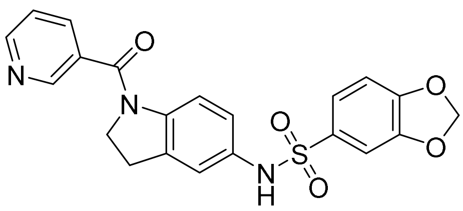
16	UL-FFA_BAC-78	*328.40*	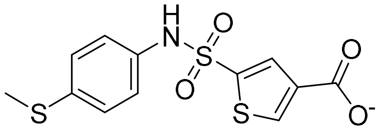
17	UL-FFA_BAC-79	*418.48*	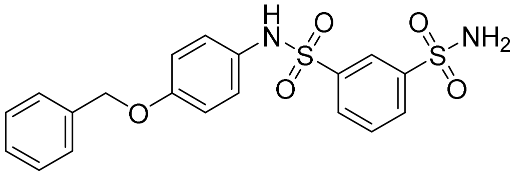
18	UL-FFA_BAC-80	*412.46*	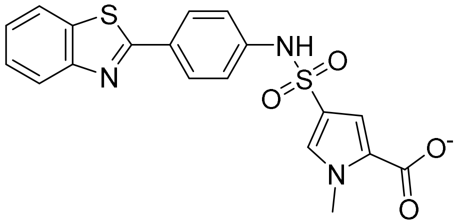
19	UL-FFA_BAC-81	*382.81*	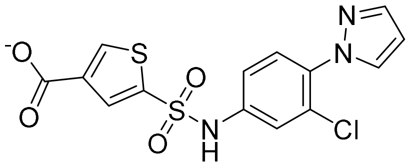
20	UL-FFA_BAC-82	*340.39*	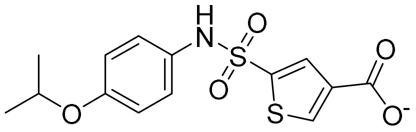
21	UL-FFA_BAC-83	*339.38*	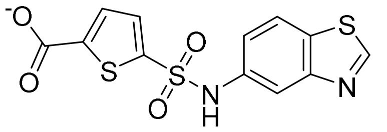
22	UL-FFA_BAC-84	*325.33*	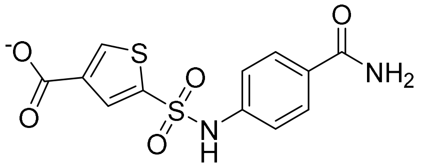
23	UL-FFA_BAC-85	*398.43*	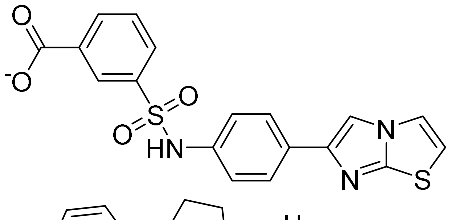
24	UL-FFA_BAC-86	*383.43*	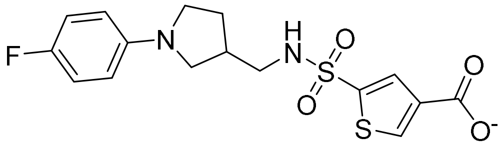
25	BAS01	310,44	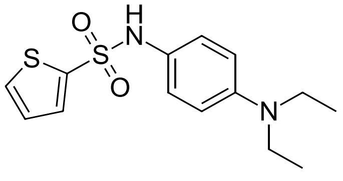

26	BAS02	330,82	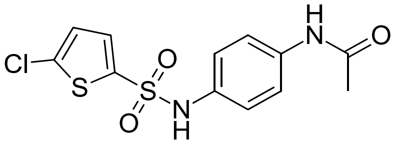
27	BAS03	287,79	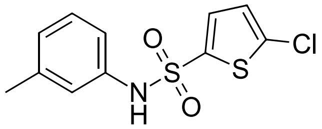
28	BAS04	360,87	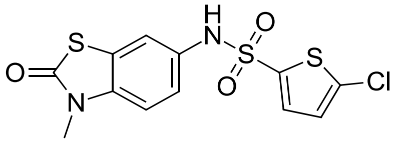
29	BAS05	312,40	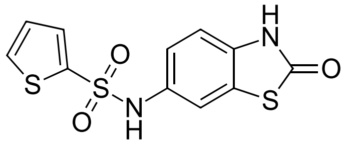
30	BAS06	346,84	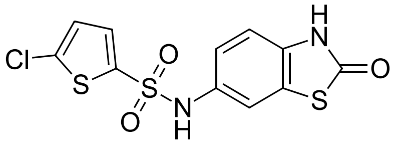
31	BAS07	323,83	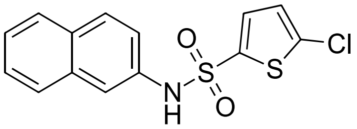
32	BAS08	289,38	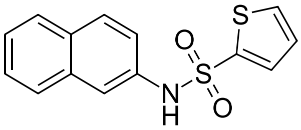
33	BAS09	338,45	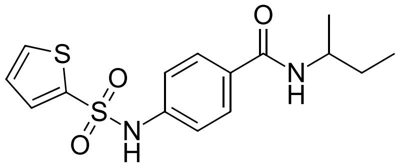

Biological Evaluation

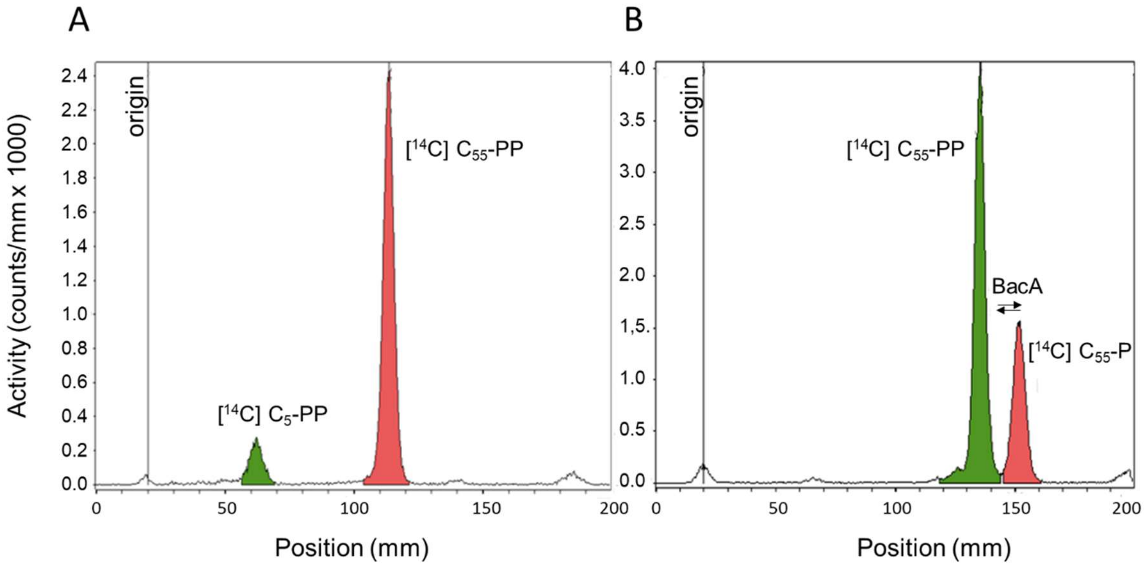

Figure S1: TLC analysis of $[^{14}C]C_{55}$-PP synthesis and $[^{14}C]C_{55}$-PP dephosphorylation. **A.** $[^{14}C]C_{55}$-PP was synthesized based on the principle of de novo synthesis via eight consecutive condensations of $[^{14}C]C_5$-PP onto C_{15}-PP, catalyzed by UppS. **B.** The transformation of the synthesized $[^{14}C]C_{55}$-PP to $[^{14}C]C_{55}$-P by BacA via in-vitro kinetic assay.

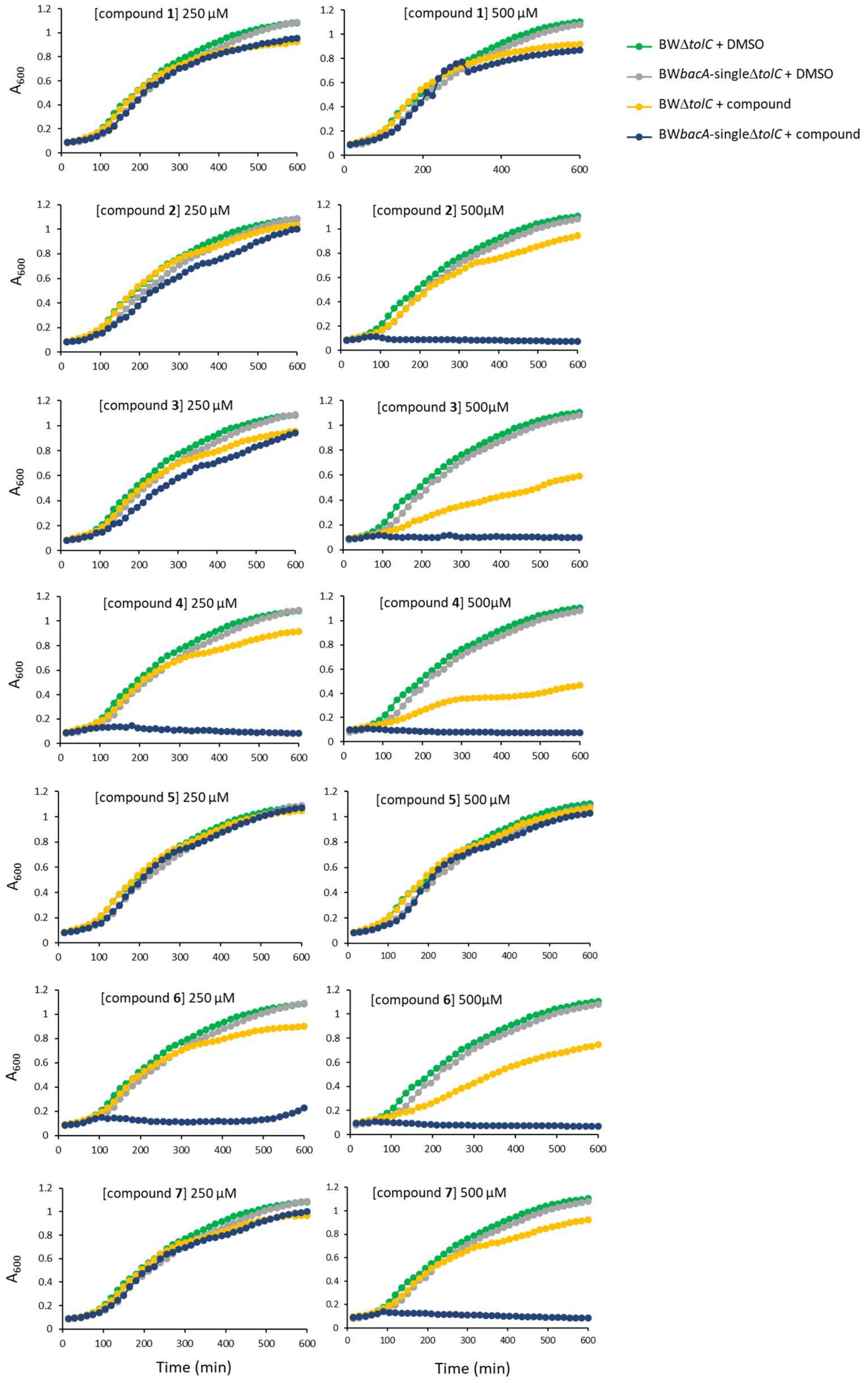

Figure S2. MIC measurements of BacA inhibitors against BWΔ*tolC* and BW*bacA*-singleΔ*tolC* strains. Representative growth curves of bacterial cells obtained in 2YT medium in the absence or the presence of inhibitors at 250 µM (left panels) and 500 µM (right panels). The added compound is specified on top of each panel. A dose of 125 µM was also tested for all compounds without any effect on growth (data not shown). We can distinguish three classes of compounds at the tested concentrations, 1) those without any antibacterial activity whatever the strain (compounds **1** and **5**), 2) those with an antibacterial activity against BW*bacA*-singleΔ*tolC* only, suggestive of a strong specificity towards BacA (compounds **2** and **7**) and 3) those with antibacterial activity on both strains suggestive of an off-target activity (compounds **3, 4** and **6**).

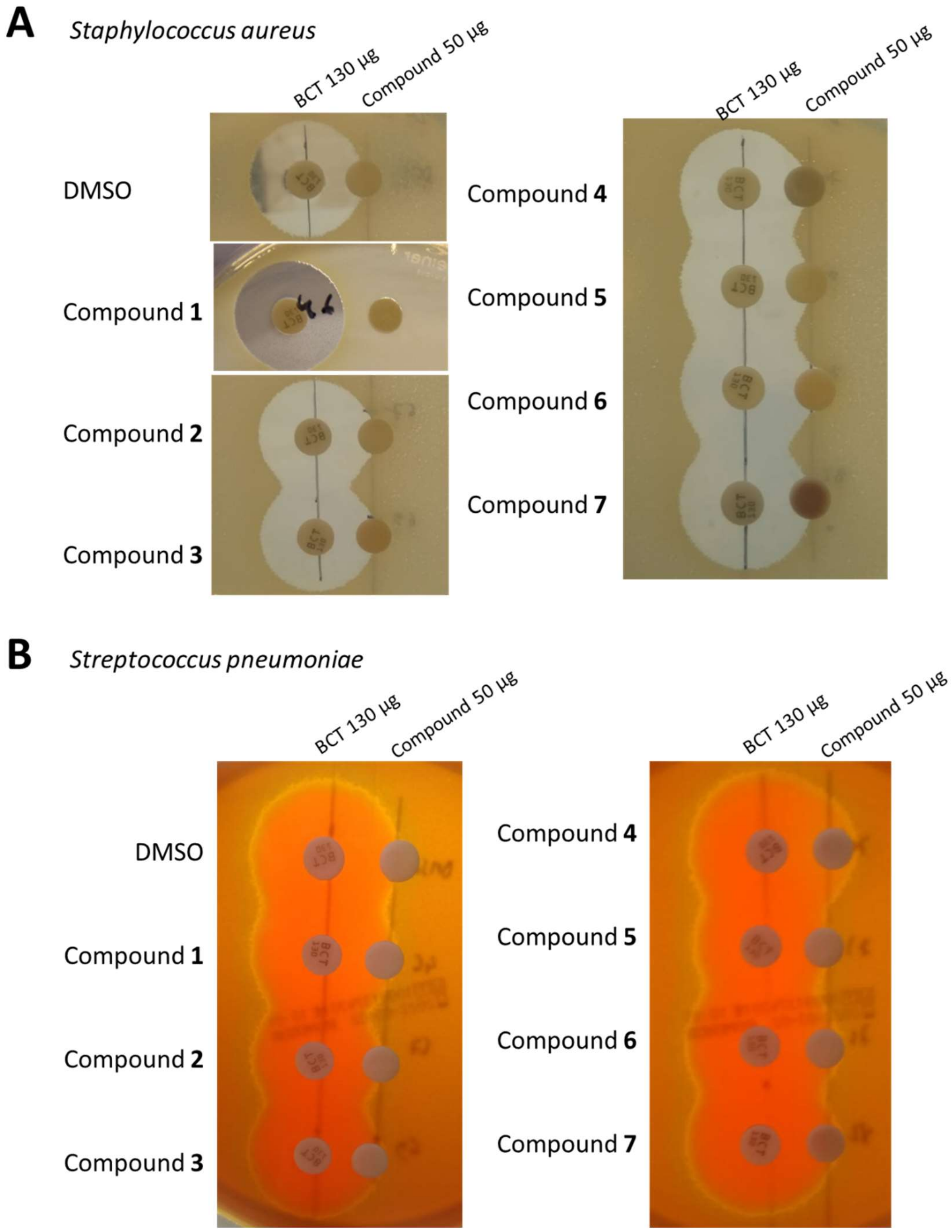

Figure S3. Test of antibacterial activity of BacA inhibitors against *S. aureus* RN4220 (A) and *S. pneumonia* R6 (B) strains. Bacitracin (130 µg)- and compound (50 µg)-loaded discs were positioned at 10 mm of distance on top of a bacterial lawn. The plates were incubated overnight at 37 °C. These images are representative of three independent tests.

Analytics

^{1}H NMR spectra were recorded in a deuterated solvent on a Bruker Avance III 400 MHz spectrometer, operating at 400 MHz frequency. NMR spectra were measured at room temperature (25 °C). Chemical shifts (δ) are expressed in *parts per million* (*ppm*) referenced to TMS or residual solvent signals. Spectral data are reported in the following format: chemical shift (multiplicity, coupling constants, number of hydrogens). All coupling constants (*J*) are reported in Hertz. HRMS mass spectra were recorded using a Thermo Scientific Q Exactive Plus mass spectrometer. Analytical reversed-phase HPLC for purchased compounds was performed on Thermo Scientific Dionex UltiMate 3000 UHPLC modular system, equipped with a photodiode array detector set to 254 nm or 220 nm. An Acquity UPLC® BEH Phenyl Column (2.1 × 100 mm; 1.7 µm) was used, it was thermostated at 40 °C, and flow rate was set to 0.3 mL/min. 1 µL of 0.5 mM sample solution in MeCN/H_2O = 4/1, V/V, was injected. An eluent system of A (0.1% TFA in H_2O) and B (MeCN) was used with gradient elution: 0–2 min 5% B; 2–7 min, 5% B → 95% B; 7–8.3 min, 95% B; 8.3–8.5 min, 95% B → 5% B.

Compound **1** (Bac46; ZINC12507960) 5-{p-[(3-Pyridyl)methylthio]phenylaminosulfonyl}-3-thenoic acid;

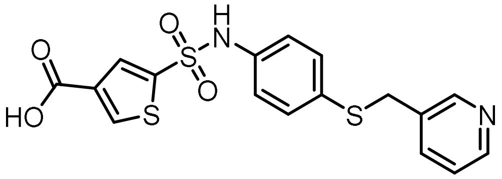

^{1}H NMR (400 MHz, DMSO-*d*$_6$) δ 10.56 (s, 1H), 8.51 (d, *J* = 1.6 Hz, 1H), 8.43 (m, 1H), 8.40 (dd, *J* = 4.8, 1.7 Hz, 1H), 7.68 (d, *J* = 1.6 Hz, 1H), 7.63 (dt, *J* = 7.9, 2.0 Hz, 1H), 7.31 – 7.24 (m, 3H), 7.09 – 7.02 (m, 2H), 4.18 (s, 2H); HRMS (ESI$^+$) *m/z* [M+H]$^+$, calcd. for $C_{17}H_{15}N_2O_4S_3$: 407.01885, found: 407.01758; Purity by HPLC: 93.4%.

Compound **2** (Bac67; ZINC15243150) 5-(3,4-Xylidinosulfonyl)-3-thenoic acid;

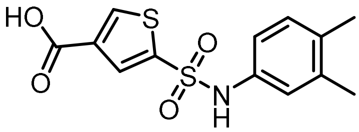

^{1}H NMR (400 MHz, DMSO-*d*$_6$) δ 13.16 (s, 1H), 10.31 (s, 1H), 8.48 (d, *J* = 1.6 Hz, 1H), 7.65 (d, *J* = 1.5 Hz, 1H), 7.04 (d, *J* = 8.1 Hz, 1H), 6.91 (d, *J* = 2.3 Hz, 1H), 6.85 (dd, *J* = 8.1, 2.4 Hz, 1H), 2.13

(d, 6H); HRMS (ESI$^-$) *m/z* $[M–H]^-$, calcd. for $C_{13}H_{12}NO_4S_2$: 310.02132, found 310.02139; Purity by HPLC: 89.2%.

Compound **3** (Bac69; ZINC13454058) 5-(1-Benzothiophen-5-ylaminosulfonyl)-3-thenoic acid;

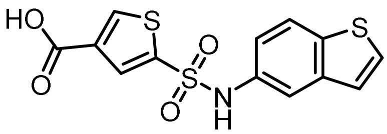

^{1}H NMR (400 MHz, DMSO-d_6) δ 13.09 (s, 1H), 10.59 (s, 1H), 8.46 (d, *J* = 1.6 Hz, 1H), 7.92 (d, *J* = 8.6 Hz, 1H), 7.78 (d, *J* = 5.4 Hz, 1H), 7.68 (d, *J* = 1.6 Hz, 1H), 7.66 (d, *J* = 2.1 Hz, 1H), 7.44 (dd, *J* = 5.4, 0.8 Hz, 1H), 7.14 (dd, *J* = 8.7, 2.1 Hz, 1H); HRMS (ESI$^-$) *m/z* $[M–H]^-$, calcd. for $C_{13}H_8NO_4S_3$: 337.96209, found 337.96232; Purity by HPLC: 90.3%.

Compound **4** (Bac70; ZINC71808762) 5-[3-Fluoro-4-(1-pyrrolidinyl)phenylaminosulfonyl]-3-thenoic acid;

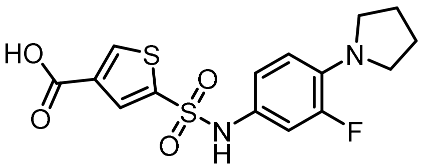

^{1}H NMR (400 MHz, DMSO-d_6) δ 13.20 (s, 1H), 10.15 (s, 1H), 8.50 (d, *J* = 1.5 Hz, 1H), 7.63 (d, *J* = 1.5 Hz, 1H), 6.84 – 6.59 (m, 3H), 3.25 (h, *J* = 2.9 Hz, 4H), 1.89 – 1.80 (m, 4H); HRMS (ESI$^+$) *m/z* $[M+H]^+$, calcd. for $C_{15}H_{16}N_2O_4S_2F$: 371.05300, found: 371.05206; Purity by HPLC: 91.1%.

Compound **5** (Bac73; ZINC72306282) 5-(2-Oxo-3H-1,3-benzothiazol-6-ylaminosulfonyl)-3-thenoic acid;

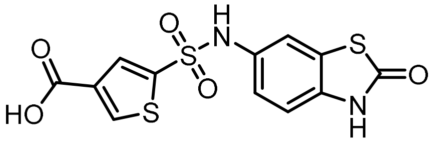

^{1}H NMR (400 MHz, DMSO-d_6) δ 13.18 (s, 1H), 11.89 (s, 1H), 10.43 (s, 1H), 8.49 (d, *J* = 1.5 Hz, 1H), 7.66 (d, *J* = 1.6 Hz, 1H), 7.35 (d, *J* = 2.1 Hz, 1H), 7.07 – 6.96 (m, 2H); HRMS (ESI$^-$) *m/z* $[M–H]^-$, calcd. for $C_{12}H_8N_2O_5S_3$: 354.95226, found 354.95237; Purity by HPLC: 94.0%.

Compound **6** (Bac76; ZINC12508252) 5-(2-Naphthylaminosulfonyl)-3-thenoic acid;

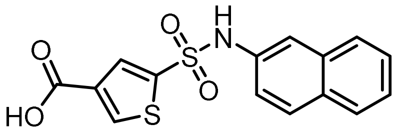

^{1}H NMR (400 MHz, DMSO-d_6) δ 13.19 (s, 1H), 10.78 (s, 1H), 8.46 (d, J = 1.6 Hz, 1H), 7.90 – 7.78 (m, 3H), 7.73 (d, J = 1.6 Hz, 1H), 7.65 (d, J = 2.2 Hz, 1H), 7.46 (dddd, J = 19.3, 8.2, 6.9, 1.4 Hz, 2H), 7.32 (dd, J = 8.7, 2.2 Hz, 1H); HRMS (ESI$^-$) m/z [M–H]$^-$, calcd. for $C_{15}H_{10}NO_4S_2$: 332.00567, found 332.00578; Purity by HPLC: 88.0%.

Compound **7** (Bac82; ZINC58370556) 5-(p-Isopropoxyphenylaminosulfonyl)-3-thenoic acid;

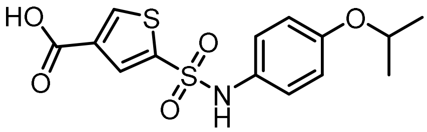

^{1}H NMR (400 MHz, DMSO-d_6) δ 13.20 (s, 1H), 10.15 (s, 1H), 8.49 (d, J = 1.6 Hz, 1H), 7.59 (d, J = 1.5 Hz, 1H), 7.04 – 6.95 (m, 2H), 6.90 – 6.80 (m, 2H), 4.51 (hept, J = 6.1 Hz, 1H), 1.21 (d, J = 6.0 Hz, 6H); HRMS (ESI$^-$) m/z [M–H]$^-$, calcd. for $C_{14}H_{14}NO_5S_2$: 340.03189, found 340.03202; Purity by HPLC: 91.8%.

Compound **8** (Bac87 ZINC91908370) N-[(3R,4R)-4-(4-Methyl-1-piperazinyl)tetrahydro-3-furyl]-3,4,7-trimethyl-1H-indole-2-carboxamide;

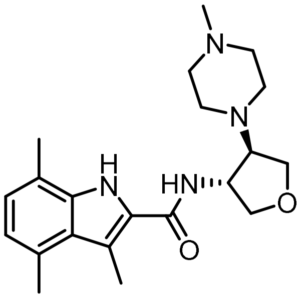

HRMS (ESI$^+$) m/z [M+H]$^+$, calcd. for $C_{21}H_{31}N_4O_2$: 371.24415, found: 371.24313; Purity by HPLC: 92.4%.
